# Field‐Induced Transparent Electrode‐Integrated Transparent Solar Cells and Heater for Active Energy Windows: Broadband Energy Harvester

**DOI:** 10.1002/advs.202303895

**Published:** 2023-07-12

**Authors:** Malkeshkumar Patel, Sangho Kim, Joondong Kim

**Affiliations:** ^1^ Photoelectric and Energy Device Application Lab (PEDAL), Multidisciplinary Core Institute for Future Energies (MCIFE) and Department of Electrical Engineering Incheon National University 119 Academy Rd. Yeonsu Incheon 22012 Republic of Korea

**Keywords:** active energy windows, deicing, field‐induced transport, transparent heater, transparent photovoltaics

## Abstract

Invisible power generation by natural and artificial light enables sustainability by onsite‐power deployment, lower cost, and minimal burden on the built environment. However, dark, opaque photovoltaics limit light utilization in a transparent way. Herein, it is proposed that the active energy window (AEW) invisibly features power production, providing higher freedom for onsite power generators in window objects without limiting human vision. The AEW has a transparent photovoltaic (TPV) for onsite power and a transparent heater (TH) to remove the effects of shadows from snow and recover the power lost. Moreover, a heating function is applied to remove the effects of weathering related to snow. The proposed prototype integrates a TPV‐TH, offering ultraviolet (UV)‐blocking, daylighting, thermal comfort, and onsite power with a power conversion efficiency of 3% (AM1.5G). Field‐induced transparent electrodes are applied to the TPV‐TH and designed considering the AEW. Owing to these electrodes, the AEW ensure a wide field‐of‐view without optical dead zones, ensuring see‐through vision. The first TPV‐TH integration is performed into a 2 cm^2^‐window that generates onsite power of 6 mW and has an average visible transmittance of ≈39%. It is believed that light can be utilized with comfort through the AEW in self‐sustainable buildings and vehicles.

## Graphical abstract

To exploit light in innovative ways, an active energy window device is presented that integrates a field‐induced transparent electrode‐embedded transparent‐photovoltaic‐heater, enabling effective power production onsite, providing daylighting to a building, and giving proper thermal comfort. Owing to its high transmittance and angular aesthetics, it can also reduce the energy used in buildings for electricity, lighting, and thermal regulation.

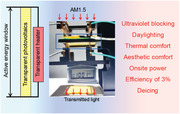

## 1 Introduction

Progressively enthralling research on transparent optoelectronics has made our lives more exciting, particularly regarding the different ways we can use such devices in our daily lives. The material of transparent electrodes (TEs) resemble the skin of devices used in both high‐tech and low‐tech applications, such as antistatic coatings, touch displays, solar cells, flat panel displays, heaters, defrosters, and optical coatings.^[^ ^1^, ^2^, ^3^, ^4^, ^5^, ^6^ ^]^ Regarding TEs in conducting oxides, metal nanowires (NWs), carbon nanotubes, graphene, organic hybrids, oxides, and their hybrids are preferred for energy devices owing to their stable transparency, electronic conductivity, and scalability in preparation.^[^ ^7^, ^8^ ^]^ The seminal work of Hosono and Ohta^[^ ^1^ ^]^ on optoelectronic devices using TEs and oxide heterostructures mentions the functional role of oxides. These devices include thin‐film transistors (TFTs) based on room‐temperature‐prepared amorphous indium gallium zinc oxide (IGZO), light‐emitting diodes with n‐ZnO/p‐SrCu_2_O_2_ heterojunctions, and UV photodiodes made of n‐ZnO/p‐NiO,^[^ ^1^ ^]^ which have emerged as unconventional platforms. The high mobility and stability of such oxides make them indispensable for use as transparent optoelectronics.^[^ ^9^ ^]^ Here, mobility refers to the efficiency of electron transport in a solid under an electric field; accordingly, a higher mobility results in a higher current, which leads to a higher performance. Regarding the orbital structure and ionic bonding of metal oxides, the *s* orbital overlaps extensively with neighboring *s* orbitals, providing a facile pathway for electron conduction. The spherical symmetry of the *ns* orbital makes the metal‐oxide material insensitive to structural distortion; therefore, oxide semiconductors have high mobility even in the mixed phase.^[^ ^10^ ^]^ This virtue of oxide materials supports the integration of onsite power harvesters with oxide electronics, paving the way for practical applications with adaptable features.

Transparent photovoltaics (TPVs) incorporate the use of solar energy with the working of components in building infrastructure, such as windows, to address energy, environmental, and sustainability issues. A TPV device (TPVD) provides electric power and intrinsic visible transparency for electric windows. This feature enables harvesting of the total solar spectrum, resulting in the generation of onsite power (bifacial) and development of see‐through devices, daylighting, aesthetics, and the ability to integrate with the existing windows of buildings, automobiles, and displays. TPVs are classified based on the average visible transmittance (AVT) value, which ranges from 20% to 80% for non‐selective or UV/near‐infrared (NIR) selective materials.^[^ ^11^, ^12^, ^13^, ^14^, ^15^, ^16^ ^]^ Non‐selective materials like perovskite‐MAPbI_3−x_Cl_x_, copper indium gallium selenide (CIGS), and a‐Si; and selective materials like organic and dye‐sensitized ones with AVT values of 25% and power conversion efficiencies >6%, have been reported with growing interest in multi‐disciplinary groups.^[^ ^16^, ^17^, ^18^, ^19^ ^]^ To realize the potential of transparent solar windows, the material of the windows should be stable, environmentally friendly, and resistant against climatic conditions that affect building envelopes. Inclusive of a transparent heater (TH), a see‐through device also consists of TEs, which generate heat via the Joule effect and are beneficial in sustainability applications such as defrosting and deicing.^[^ ^20^ ^]^ TEs can be used in integrating TPV and TH devices, eventually leading to the development of energy windows that offer city‐integrated renewable energy for utilizing full‐spectrum solar energy. They are also irrefutably useful as components in building self‐sustainable cities.^[^ ^17^, ^21^, ^22^ ^]^

The glass window is a large building material for the cities and has the potential for building sustainable cities. In this order, windows should be energy generators with energy densities of 20−50 µW cm^−2^ for smart windows, 2−20 mW cm^−2^ for buildings, and 1.2−45 mW cm^−2^ for electric vehicles.^[^ ^14^ ^]^ Such power‐generating windows open avenues for distributed applications due to easy operation and a lower balance of system costs.^[^ ^23^ ^]^ A recent study shows the energy‐efficient and self‐sustainable management of buildings by using dynamic photovoltaic employment.^[^ ^24^ ^]^ Even more, active control schemes of buildings have been performed by daytime radiative cooling and solar heating for year‐round energy saving.^[^ ^25^ ^]^ Further, triboelectric nanogenerators harvest waste energy from utilities and provide electricity for buildings and transportation systems.^[^ ^26^, ^27^ ^]^ Furthermore, raindrops also facilitate electricity generation through windows for building electricity.^[^ ^28^, ^29^, ^30^ ^]^ Various approaches to energy window power applications are summarized in **Table** **1**.

**Table 1:** Summary of energy power window prototypes

Energy window prototypes	Components	Transparent	Feature	Remarks	Reference
Dynamic photovoltaic building envelop	PV panel, pneumatic control module, 2‐axis soft actuator,	Partially transparent	Increase 50% electricity gains	Complex and dynamic operation. No thermal control.	[24]
Radiative cooling and solar heating	PI film, Cu‐Cu/Zn for heating function, Ag/PDMS for cooling function	No	Thermal control for low and cold climate	Complex and dynamic operation.	[25]
Droplet‐based electric window	Glass, ITO, AgNW, PTFE, Pt, Electronic circuit	No	Power generation with high voltage	Rain is needed, power output depends on raindrop velocity. No thermal control.	[29]
Triboelectric nanogenerator‐based electric window	Silicon rubber ball, Cu electrode, PLA matrix, TENG‐structure, electronic circuit	No	Power generation with high voltage and density	Mechanical energy is needed as input energy. No thermal control.	[26]
Luminescent solar concentrator solar window	Carbon dots, Ag particle, Ag/SiO_2_ core‐shell particle, mirror, high‐performance PV cell array	Yes	Onsite power with color control	Complex assembly, bulky module. No thermal control.	[31]
Selective Solar Harvesting Windows	Transparent photovoltaic, ventilation, PV cells, transparent solar absorber, E‐glass frame	Yes	Electricity and thermal control with ventilation	Complex system, ventilation for thermal control, heavy structure.	[4]
Active energy window	AgNW, ZnO, AZO, AgNW, a‐Si, glass, Cu wire	Yes	UV blocking, Onsite power, Thermal‐visual comfort, Deicing	Easy‐function, available as building materials, power density 3 mW/cm^2^	This study

Herein, we report an active‐energy control window that integrates a TPV and TH through field‐induced transparent electrodes (FITEs). The TPV‐TH module can generate electric power from light and utilize this power to regulate energy for buildings. The properties and performances of each device regarding their thermal durability, seasonal variation, and deicing were investigated. We then integrated these devices as prototype applications in buildings for solar‐energy utilization and weatherization. A state‐of‐the‐art setup was used to demonstrate the performance of the TPV‐TH integrated device with respect to onsite power generation and stability. We also demonstrated onsite power production by operating a fan with the integrated TH‐TPV module under a solar simulator having an intensity of 100 mW cm^−2^ and power loss due to snow accretion on the TPV‐TH roof. Then, we performed deicing using the TH, which recovered the lost power, supported daylighting, and provided thermal comfort.

## 2 Results and Discussion

### 2.1 Field‐Induced Transparent Electrode (FITE) for Broadband Energy

**Figure 1:**
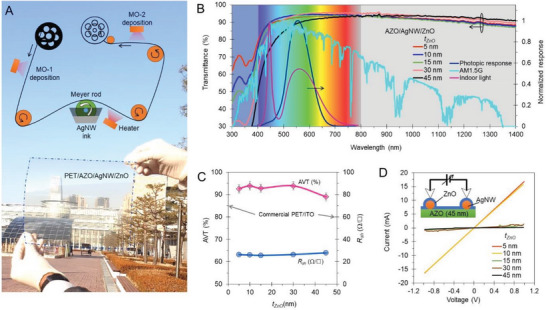
Field‐induced transparent electrode (FITE) with oxide heterostructure and metal nanowire (NW) composite. A) Fabrication process and original sample of transparent electrode (TE) consisting of zinc oxides (ZnOs) and silver nanowire (AgNWs). B) Transmittance profile of AZO/AgNW/ZnO samples, photopic response and light irradiance spectra of AM1.5G, and indoor white light‐emitting diode (LED) at 6000 K. C) Average visible transmittance (AVT) and sheet resistance (*R*_sh_) as a function of the thickness of the top ZnO film (*t*_ZnO_). D) Current‐voltage characteristics of TEs having top ZnO films with thicknesses ranging from 5 to 45 nm (inset in schematic shows measurement setup with an electrode spacing of 1 cm).

TEs integrate oxide heterostructures and silver nanowires (AgNWs); these NWs are fabricated and grown in large areas under two processes, namely magnetron sputtering and Meyer rod coating. This approach supports the nanoscale thickness control of these NWs, which can improve their optical, electrical, and interfacial properties.^[^ ^6^ ^]^ **Figure** **1**A shows the steps in the fabrication procedure used to produce TEs of an AZO/AgNW/ZnO composite on a polyethylene terephthalate (PET) substrate with a size of 500 cm^2^. In the design, an oxide heterostructure of intrinsic ZnO and Al‐doped ZnO (AZO) was employed to induce an electric field using the difference in their net doping concentrations and their integration through the AgNW electron cloud. This design was referred to as the FITE. Figure 1B shows the transmittance profiles of the FITEs with an AgNW network with bottom AZO and top ZnO films that were assembled considering light irradiance and the photonic responses of the human eye. This result showed the controllable broadband transmittance in the UV, visible, and infrared (IR) regions of the top ZnO films. However, an FITE with broadband transmittance is preferred in processing the broadband solar spectrum. In contrast to indoor light‐emitting diode (LED) light, which covers blue and red wavelengths, the AM1.5G irradiance of 1000 W m^−2^ was distributed over the UV region from 280 to 400 nm of 93.9 W m^−2^, the visible region from 400 to 800 nm of 496 W m^−2^, and the NIR region from 800 to 1400 nm of 353 W m^−2^. Owing to the broadband transmittances among various TEs, an FITE can be applied to total‐spectrum energy‐harvesting devices.^[^ ^8^ ^]^ To find the suitable thickness of the top ZnO film (*t*_ZnO_), the AVT (described in the section of Aesthetics assessment) and sheet resistance (*R*_sh_) for *t*_ZnO_ were assessed, as shown in Figure 1C; for *t*_ZnO_ ≈10 nm, there are AVT and *R*_sh_ values of 93% and 26 Ω cm^−2^, respectively, which are superior to those of commercial PET/indium tin oxide (ITO) (Sigma–Aldrich, 639 303).

We prepared two reference sets to check and confirm the possibility of electric transport in the designed FITE. In the first case, a 45 nm ZnO base layer grown on glass was applied to the AgNW and top ZnO. In the latter case, 45 nm of the AZO base layer was applied to the AgNWs and top ZnO film; in both cases, the *t*_ZnO_ varied from 5 to 45 nm. Figure 1D shows the current‐voltage characteristics of the AZO/AgNW/ZnO used in designing the FITE (samples and current measurement setup is shown in Figure S1, Supporting Information). There was a consistent current of 16 mA in the *t*_ZnO_ range of 5–10 nm; when *t*_ZnO_ >15 nm, the current substantially decreased to 0.5 mA. In contrast, the ZnO/AgNW/ZnO sample showed a consistent current value of 15 mA for a *t*_ZnO_ of 5–30 nm and then abruptly dropped to 2.5 mA for a *t*_ZnO_ of 45 nm (Figures S2 and S3, Supporting Information). These results revealed distinct electric transports, which were attributed to the carrier confinement by the field induction at ZnO/AZO and the integration of ZnO/AZO at the AgNW interfaces.

**Figure 2:**
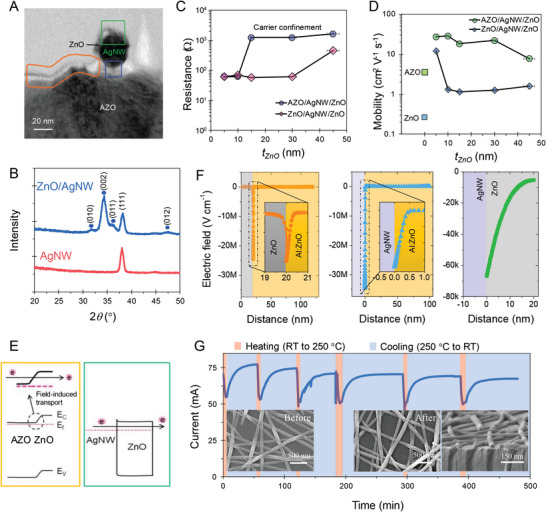
Electrical transport mechanism of the AZO/AgNW/ZnO FITE. A) Cross‐section of the ZnO/AgNW/AZO specimen, where the electric field induction by the AZO/ZnO interface is marked in orange, the merging of the electric field with the electron cloud of AgNW is marked in blue, and the ZnO/AgNW interface is marked in green. B) X‐ray diffraction (XRD) plots (blue circle shows diffraction peaks of the ZnO). C) Resistance versus *t*_ZnO_ measured at ±1 V. D) Electron carrier mobility of AZO, ZnO, and their electrodes as a function of *t*_ZnO_. E) Energy band diagram of AZO/ZnO interface shows the electric field, and the AgNW/ZnO interface shows the tunnel‐assisted electron transport of ZnO. F) Electric field formation at ZnO/AZO, AgNW/AZO, and AgNW/ZnO interfaces. Inset shows electric field profiles at the interface as a function of distance. G) Current profile of the AZO/AgNW/ZnO electrode under transient thermal conditions. The inset shows the cross‐section and topography of the specimen before and after the test. Regions corresponding to the heating from room temperature (RT) to 250 °C and cooling from 250 °C to the RT are marked.

**Figure** **2**A shows a cross‐sectional view of the AZO/AgNW/ZnO sample (obtained using bright field mode), which can clarify the FITE structure; the AZO/ZnO, AZO/AgNW, and AgNW/ZnO regions are also shown. An AgNW with a diameter of 20 nm, intact with 10 nm of ZnO, can be observed on the AZO film. A layer of ZnO was uniformly coated on the AZO and AgNW. Figure 2B shows the X‐ray diffraction (XRD) pattern of the AgNW and that the AgNW/ZnO confirms the single‐crystal cubic structure of the AgNWs with the (111) plane and nanocrystalline structure of the hexagonal crystal system of the ZnO film (represented by blue circles shows *hkl* indices of (010), (002), (011), and (012)).

The electric transport of the FITE‐based TEs can be confirmed by observing the changes in resistance, as shown in Figure 2C. The resistance values of both the sets of electrodes with various *t*_ZnO_ values can be observed consistently within 5 and 10 nm; the resistance abruptly increased to a few kΩ for *t*_ZnO_>15 nm, which was attributed to the carrier confinement at the ZnO/AZO interface (Table S1, Supporting Information). Resistance values were simultaneously observed for the ZnO/AgNW/ZnO electrodes with *t*_ZnO_ values ranging from 5 to 30 nm. This observation suggested that the confinement carried by the ZnO/AZO interface could be achieved through thickness tuning. The mobility of confined electrons can be much higher, owing to the reduced scattering and facile pathways, which have attracted significant attention.^[^ ^32^, ^33^, ^34^ ^]^ Figure 2D shows the Hall mobilities of both electrodes with varying *t*_ZnO_ values. Higher and consistent mobility values were recorded for the AZO/AgNW/ZnO–FITE structure, where *t*_ZnO_ values of 5 and 10 nm provided mobility values of 28.7 cm^2^ V^−1^ s^−1^, which was notably higher than that of the ZnO and AZO films individually. The Hall measurement samples are shown in Figure S4 (Supporting Information).

### 2.2 Mechanism behind Electrical Conduction of FITE

Conduction through a metal probe and the surface of a material significantly changes with the potential distance and a slight change in the barrier width.^[^ ^35^ ^]^ Under the tunneling phenomenon, the transmission coefficient for conduction electrons in the AZO/AgNW/ZnO sample depends on *t*_ZnO_. For a wide or high barrier, the relative probability that electrons are confined in the tunnel can be measured using the transmission coefficient *T*, which depends very strongly on the height (*V*_o_‐*E*) and width *Γ* of the potential barrier; it can be expressed using the following relations:

(1) $$T=T_{o}\exp\left(-2\alpha \Gamma \right)$$

(2) $$T_{o}=\frac{16\left(V_{o}-E\right)}{V_{o}^{2}}$$

where α is the quantized energy in a finite quantum well, *V*_O_ is the potential energy barrier, and *E* is the kinetic energy of an electron.^[^ ^35^ ^]^ Figure 2E shows the energy band diagram of AZO/ZnO for field formation and that of AgNW/ZnO for electron conduction through tunneling (Figure S5, Supporting Information, shows the energy band edges of AZO, AgNW, and ZnO). The thickness of the top ZnO film, *t*_ZnO_, plays a crucial role in electron conduction in FITE, resulting in a lower sheet resistance, higher mobility, and broadband transmittance.^[^ ^8^ ^]^

It is useful to establish the electric field formation as to how electron carriers transport the energy bandgap. Three cases were investigated for ZnO/AZO, AZO/AgNW, and ZnO/AgNW interfaces. A 1D Poisson equation was performed by the SCAPS simulation.^[^ ^36^ ^]^ The results are graphically presented in Figure 2F. The strongest electric field value is found from the AZO/AgNW interface to be 3.46 × 10^7^ V cm^−1^. Interestingly, similar high electric field is formed in the interface of ZnO/AZO by 2.45 × 10^7^ V cm^−1^. Meanwhile, the moderate value of electric field (6.62 × 10^4^ V cm^−1^) is generated between ZnO and AgNW interface. This is attributed to the lower doping concentration of the ZnO layer (10^17^ cm^−3^), resulting in the broader electric field distribution (>15 nm, Figure 2F). In comparison, very sharp and narrow electric field profile is traced for the ZnO/AZO interface (<2 nm). For the Schottky‐like interface of AgNW/AZO, cutting‐edge shaped electric field distribution (<1 nm) is formed. This calculation is complementarily evident in the energy‐band diagram shown in Figure 2E, where the efficient electron collection at ZnO/AZO and the tunnel mechanism through top ZnO are correspondingly illustrated. This clearly indicates that the electric‐field assisted transport mechanism is effective for the heterostructure of AZO/AgNW/ZnO to have a higher mobility value, compared to the case of ZnO/AgNW/ZnO.

### 2.3 Thermal Stability of FITE

The thermal stability of a device is important when considering its practical applicability; it has attracted significant research attention recently. Compared to bare AgNWs and other emerging TEs, the AZO/AgNW/ZnO sample with the FITE structure can offer good thermal stability because of the top ZnO layer.^[^ ^5^, ^8^ ^]^ To verify the thermal stability, a transient thermal condition (ramping up to 250 °C followed by quenching) was applied for 12 h, as shown in Figure 2G. The sole AgNWs exhibited substantial morphological changes that could be inferred from the time function. In the initial stage, the AgNWs physically overlapped and formed networks. As time passed, the thermal stress accumulated inside the AgNWs caused the Rayleigh instability stage to induce a drastic increase in the resistance at a specific time. Later, there was severe deformation in the NWs, resulting in network loss following spheroidization that stabilized droplets with low free energy under thermal processes. Transient thermal measurement for the AgNW electrode shows the susceptible current profile of the AgNW network to thermal stimuli (Figure S6a, Supporting Information). The resistance with bias of +0.5 V was ≈10 Ω in the beginning, which approached >10 kΩ in 4 min of thermal cycle. The corresponding change of AgNW morphology (in Figure S6b, Supporting Information) shows various phases of deformation, elucidates the loss of the conduction network.

In contrast, the AgNWs with the FITE structure exhibited stable electrical transport under thermal stress. Figure 2F shows the prolonged current flow through the FITE under cyclic thermal stress. The current was reduced from 70 to 55 mA during the heating interval. Following quenching, it returned to ≈75 mA (the magnified heating and cooling cycles are shown in Figure S7, Supporting Information). The topography of the FITE samples after being subjected to cyclic thermal stress is shown in the inset of Figure 2G. Under steady and transient thermal stress cycles, the current flowing through the AZO/AgNW/ZnO electrodes and the preserved structure demonstrated the superior thermal stability of the FITEs. Hence, we applied AZO/AgNW/ZnO–FITE‐based TPV and TH devices and their integrated modules; this is discussed in the following section.

### 2.4 FITE‐Integrated TPV Cells

**Figure 3:**
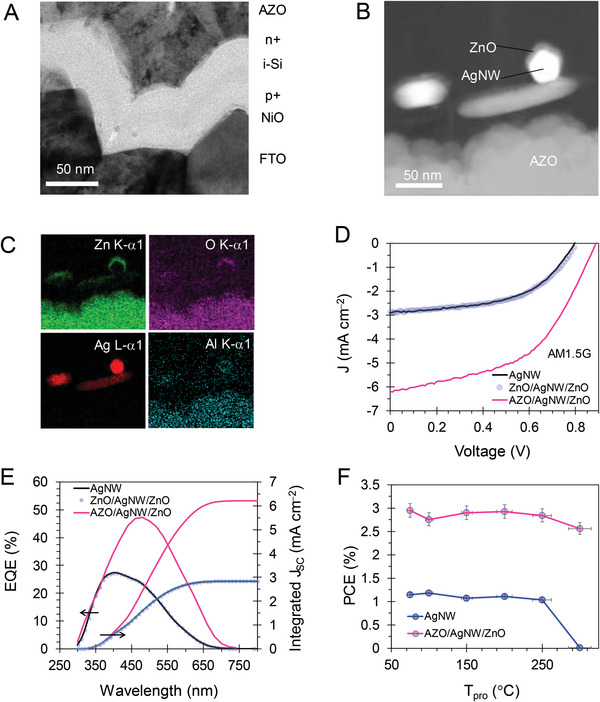
Structure and performances of the transparent photovoltaic device (TPVD) with FITE. Cross‐section of the device A) focused on the junction region of a‐Si and B) the top electrode of AZO/AgNW/ZnO. C) Elemental mapping of a top electrode showing the distribution of Zn, O, Ag, and Al. D) Current density–voltage (*J–V*) characteristic plots of devices with AgNW and ZnO/AgNW electrodes under the standard solar simulator (AM1.5G, 100 mW cm^−2^). E) External quantum efficiency (EQE) versus wavelength and integrated *J*_SC_, confirming performance compliance. F) Power conversion efficiency (PCE) versus processing temperature (*T*_pro_) showing performance of the device under thermal stress.

Durable FITE electrodes can be used as the top electrodes of TPV cells, including materials such as perovskites, organic materials, dyes, quantum dots, and inorganic thin films (a‐Si, CIGS, and CdTe).^[^ ^37^, ^38^ ^]^ Among these candidates, ultrathin a‐Si can be applied to realize the TPV‐TH device because of its eco‐friendliness, abundance, and low cost, owing to well‐established processing techniques.^[^ ^39^ ^]^ We used an ultrathin a‐Si absorber (p‐i‐n junction) having a top FITE. The device has ≈40 nm of a‐Si, revealing the conformal fabrication on SnO_2_:F (FTO), as shown in the cross‐section of the device in **Figure** **3**A. Figure 3B shows the compact arrangement of AZO/AgNW/ZnO; the FITE structure of the TPVD was clearly provided (obtained using dark field mode). The elemental distribution of Zn, O, Ag, and Al shown in Figure 3C illustrates the distribution of AgNWs on AZO. The top ZnO induced an electric field and protected the network of AgNWs and the ZnO/AZO interface for electric charge collection.

The TPVD integrated with the FITE and other top electrodes (active area of 3 cm^2^) was tested under a standard AM1.5G illumination of 100 mW cm^−2^. The *J–V* characteristics of the TPVs are shown in Figure 3D, and the performance parameters are listed in **Table** **2**. The FITE‐embedded TPV exhibited a decent power conversion efficiency (PCE) of 3.07% owing to a significantly enhanced short‐circuit current density (*J*_SC_) of 6.21 mA cm^−2^, an open circuit voltage (*V*_OC_) of 890.2 mV, and a fill factor (FF) of 55.56%. The practical onsite power production by the TPV for a moving fan is demonstrated in Video S1 (Supporting Information). The effect of the FITE was more pronounced than that of the TPVD with the AgNW and ZnO/AgNA/ZnO‐TE, which exhibited a PCE value of ≈1.2% owing to a comparatively lower *J*_SC_ of 2.8 mA cm^−2^. As noted, the parasitic element of shunt resistance (*R*_SH_) significantly improved from 245 to 800.9 Ω cm^−2^; this improvement in the junction properties was attributed to the FITE design.^[^ ^6^ ^]^ This result confirmed the onsite power generation density of 30.7 W m^−2^ with an AVT value of 43.2% using the FITE‐embedded TPVD.

**Table 2:** Performance parameters of the transparent photovoltaic device (TPVD) measured under the standard solar simulator with an AM1.5 intensity of 100 mW cm^−2^. The active area and temperature of the device are 3 ± 0.01 cm^2^ and 300 ± 0.5 K, respectively

Parameters	AgNW	ZnO/AgNW/ZnO	AZO/AgNW/ZnO
*V*_OC_ [mV]	797.34	804.89	890.2
*J*_SC_ [mA cm^−2^]	2.89	2.847	6.21
FF [%]	52.3	53.47	55.56
Efficiency	1.21	1.23	3.07
*R*_S_ [Ω cm^−2^]	25.9	22.7	18.2
*R*_SH_ [Ω cm^−2^]	245.1	365.5	800.9
AVT [%]	40.2	43.5	43.2
LUE [%]	0.484	0.533	1.33

FF‐ Fill factor

AVT‐ Average visible transmittance

LUE‐ Light utilization efficiency.

Further, the overall systematic utilization factor has been investigated in the concept of light utilization efficiency (LUE). This is the product of AVT and PCE,^[^ ^14^ ^]^ to indicate the value of light transmission through TPV while generating power. In order to view the FITE effect on LUE performances, three kinds of electrodes were deployed on the identical TPV devices, having a large‐scale of 3 ± 0.01 cm^2^ (Table 2). The sole AgNW electrode induces the LUE value of 0.484%. A certain improvement was achieved in the case for ZnO/AgNW/ZnO electrode by 0.533%. A significant enhancement (LUE of 1.33%) is established on AZO/AgNW/ZnO structure of FITE design. Due to the inherent transparency, TPV device exhibits the excellent bifacial photovoltaic performances (Figure S8, Supporting Information). Bifacial TPV shows almost identical power generation profiles. Front illumination gave a PCE of 3.07% with high *V*_OC_ (890.2 mV). In comparison, back illumination generated 2.97% of PCE, resulting in good value in bifacial ratio (PCE_front_/PCE_back_) of 1.034.

To assess the spectral performance attributes,^[^ ^40^ ^]^ the external quantum efficiency (EQE) of the transparent photovoltaic devices (TPVDs) was analyzed, as shown in Figure 3E. The response in the high‐energy wavelength (UV and blue) can be attributed to the p‐i‐n junction experiencing synergetic interaction with FITE. The lower EQE in the longer wavelength region was due to the reduced absorption and transparency of the device (Figure S9, Supporting Information). To confirm the photon balance for the AM1.5G spectrum, we estimated the *J*_SC_ value using EQE and solar spectral irradiance according to the following relation:

(3) $$Integrated\;J_{\mathrm{SC}}=q\times \int_{280\;\mathrm{nm}}^{1400\;\mathrm{nm}}\left(EQE\left(\lambda \right)\times AM1.5G\left(\lambda \right)\right)d\lambda$$

where *q* and *λ* are the unit charge and wavelength, respectively.^[^ ^40^ ^]^ The integrated *J*_SC_ value of 2.75 and 6.2 mA cm^−2^ for the AgNW and FITE‐embedded TPVDs, respectively, complied with the measured *J*_SC_, showing consistency in the results.

The EQE coverage in blue photon wavelength of the TPVD matches well to the indoor LED lighting. The setup of TPV measurement is designed for the uniform LED lighting on the see‐through TPVD arrangement. Under the LED illumination (5800 K) of 100 mW cm^−2^, the TPV device with FITE electrode exhibited excellent photovoltaic performances giving the *V*_OC_ of 922.64 mV and the *J*_SC_ of 25.03 mA cm^−2^ (Figure S10, Supporting Information). The high *J*_SC_ value is attributed to the spectral match of the LED and the reactive quantum efficiency of the TPV device, corresponding to the LED light wavelength. The relatively high power conversion efficiency (PCE) value of 9.1% was achieved from the LED lighting, which is 296.42% higher value than that of the illumination of a solar simulator (McScience AAA grade) at a certain light power density (100 mW cm^−2^). This strongly suggests the significant potential of TPV as the indoor power generator and versatile ubiquitous energy utilization.

Its integration with glass/building materials is often thermally processed to use FITE‐TPVs in buildings; therefore, its durability can be examined.^[^ ^3^, ^4^, ^22^ ^]^ This was checked by performing thermal processing in an air atmosphere for 10 min, followed by natural cooling. The AgNW‐ and FITE‐embedded TPVDs were preferred for comparison. Figure 3F shows the PCE of the TPVD as a function of the processing temperature *T*_pro_. The TPVD with the FITE electrode showed a consistent PCE in the range of 2.5–3% under all the thermal treatments.

In contrast, the PCE of the TPVD with the AgNW electrode gradually reduced and ended at a low value indicating failure for *T*_pro_ value >200 °C. Owing to the loss of the AgNW network under thermal stress, such failure also resulted in a *J*_SC_ loss due to the partial collection of the photogenerated electrons; however, the *V*_OC_ of the device was invariant under the thermal process (Figure S11, Supporting Information). This result demonstrated the durability of the TPVD (both junction and top electrode of FITE); its stable performance under harsh conditions mitigated the level of concern regarding the thermal processing ability of integrated applications in buildings.

### 2.5 Evaluation of FITE‐Embedded TPV‐TH of Active Energy Window (AEW)

**Figure 4:**
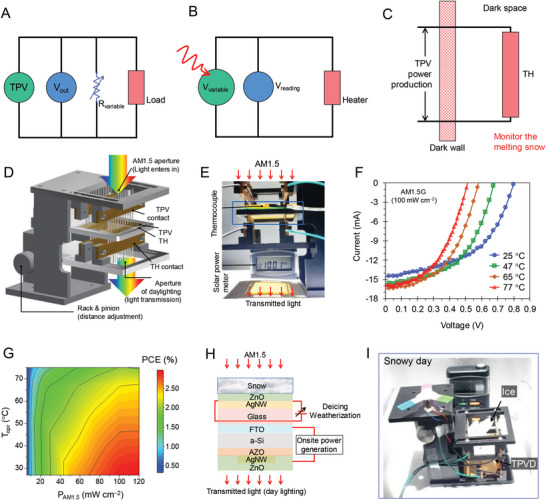
Design and assessment of active energy window (AEW). The equivalent circuit depicts the A) onsite power generation using the TPVD, B) onsite power deployment using the TH for thermal comfort, and C) melting of snow and onsite power recovery using the TPV‐TH–integrated AEW. D) Design showing light entering through AM1.5 aperture of TPV‐TH. E) TPV‐TH system under the standard solar simulator. F) *J–V* characteristic plots of TPV‐TH under irradiance of AM1.5 and operational heater. G) Contour mapping of the PCE for operational temperature (*T*_opr_) and light intensity (P_AM1.5_). H) Schematic showing onsite power generation, deicing, and daylighting using FITE‐embedded TPV‐TH system. I) Onsite power production and operation of fan using TPV‐TH under the solar simulator (100 mW cm^−2^) after snowing and loss in power generated onsite by reflections of the light from precipitated snow/shadows, onsite power production recovery through TH‐driven deicing, and recovery of power generated onsite and daylighting using a clear transparent roof (see Video S1, Supporting Information).

The active energy window (AEW) is an emerging technology that requires adherence to rigorous performance standards such as aesthetics, temperature, and light intensity variations for use in integrated and distributed building applications.^[^ ^14^ ^]^ The onsite power production for variable loads operated by TPV with an integrated FITE top electrode is depicted using a simple illustration in **Figure** **4**A. To mitigate the impact of environmental parameters and improve its integration with the building, the TH was regulated by a variable voltage source (*V*_ariable_), as depicted in Figure 4B. The AEW integrated both the TPV and TH units to replace the buildings' dark walls to recover onsite power and monitor the melting of snow, as depicted in Figure 4C. Evaluating the performance of such an AEW requires an assessment of its adherence to the aforementioned practical criteria. Yeng et al.^[^ ^40^ ^]^ demonstrated a protocol highlighting background effects, color rendering, the average transmittance of visible photons, and a consistency check for photon balance. The TH‐embedded TPVD provides an opportunity to accurately examine the performance of TPVs considering the effect of temperature, which is beyond the scope of the existing protocol. Figure 4D shows a high‐quality design for measuring the TPV with the embedded TH. Here, the adaptable contact arrays connect the TPV (front) and TH (back) through a rack‐and‐pinion assembly, resulting in high‐throughput measurements with greater accuracy. The AM1.5 aperture is defined as an opening through which external light enters to interact with solar/indoor light; daylighting is performed using such apertures, transmitting visible/IR light to the background. Contact through the pogo‐pin arrays of the top for the TPV and bottom for the TH could regulate the temperature through a pathway of the TH. Owing to the thermal and electrical insulation properties of black Delrin, a jig made of this material was prepared for the TPV‐TH system of 50 × 50 mm^2^.

The thermal performance of FITE‐based by Joule heating effect is examined comparatively with AgNW/ZnO and ZnO/AgNW/ZnO designs (Figure S12, Supporting Information). The transient thermal response of these large‐area heaters, measured with the state‐of‐the‐art setup, revealed the role of the electric field in enabling excellent thermal performances. Noteworthy to mention that the FITE electrode allowed greater current flow due to lower resistance by field effect enhanced electron transport, efficiently driving 125.5% more Joule heating performance of the FITE‐TH compared to the AgNW/ZnO TH.

Tests were performed for the variations in temperature and light intensity in a see‐through device, as shown in Figure 4E. The FITE‐embedded TPV‐TH device enabled onsite power generation with a see‐through light beam, with the TH controlling the device temperature (Figure S13, Supporting Information). The temperatures from 25 to 77 °C and light intensities from 3 to 120 mW cm^−2^ were regulated to obtain the *I–V* characteristics of the TPV‐TH for window applications (Figures S14 and S15, Supporting Information). In this temperature range, under 1 sun, the TPVD exhibited increased *I*_SC_ from 14.49 to 16.24 mA and reduced *V*_OC_ from 0.798 to 0.518 V, supporting onsite power generation at an even higher temperature (Figure 4F). This trend can be explained by the following:

(4) $$V_{\mathrm{OC}}\left(T\right)=\frac{kT}{q}\ln\left(1-\frac{J_{\mathrm{SC}}}{J_{S}}\right)$$

where *kT*/*q* and *J*_S_ are the thermal voltage and dark saturation currents, respectively.^[^ ^41^ ^]^

The contour plot for the PCE of the device in Figure 4G simply presents the relationship between the temperature and light intensity of AM1.5; this result supported power generation under operational conditions. The temperature and light intensity regulating the performance parameters of the AEW are discussed in the later section.

### 2.6 Deicing and Onsite Power Production Using AEW

Due to the merits of independent power production through the TPV, the power supply can be supported at the site of power generation, liberating the “grid” of the power‐link. Various meteorological phenomena produce deposits of water and ice on the surfaces of building envelopes worldwide, with snow being the most pronounced surface by a significant level. In countries such as Sweden, Norway, Canada, and Russia, which are in subtropical and northern regions, there are more mean snow‐cover days, that is, ≈100, and there are even habitats with 300–365 of mean snow‐cover days.^[^ ^22^, ^42^, ^43^, ^44^, ^45^, ^46^, ^47^, ^48^ ^]^ Photovoltaics (PVs) have been deployed significantly in these regions due to their reduced cost; however, due to ice precipitations, using PV panels has produced annual power losses from 15% to 34%.^[^ ^42^, ^44^, ^49^ ^]^ Snow‐related energy losses account for power losses of 35% (annual) and 70% (snow season) for PV modules with a tilt angle of 0° (Calumet, MI, USA); these losses can slightly be reduced to 30% (annual) and 60% (snow season), respectively, with a tilt angle of 35°.^[^ ^45^ ^]^ Further, precipitated snow creates an albedo effect, reflecting a significant amount of solar radiation into the atmosphere. Transmittance rapidly decreases with an increase in the snow depth (decrease in transmittance from 90% to 5% for increase in snow depth from 0.2 to 2 cm).^[^ ^42^ ^]^ Issues related to snow and ice include limitations in operational and structural design, impact of snow loads, drainage of snowmelt, and difficulties in the safe removal of snow. Exploring the use of the FITE‐based TPV‐TH AEW in providing solutions to these issues through onsite power production, daylighting, and removal of snow/precipitates in extreme climates, is highly valuable (Figure S16, Supporting Information).

A schematic of onsite power generation and deicing using the TPV‐TH device is shown in Figure 4H. A large‐area TPVD provides onsite direct current (DC) and allows daylighting and thermal comfort through the solar penetration of the building envelope. When the working of the TPVD is hampered by snow precipitations, deicing is performed by the TH, recovering the features mentioned above. The performances of various utilities such as batteries and heaters are supported by onsite clean energy and daylight, both of which significantly contribute to the energy efficiency of buildings.

Figure 4I shows the use of the FITE‐embedded TPV‐TH AEW for onsite power production, ice precipitation, and deicing. The real‐time operation of the TPV‐TH device was demonstrated, which included onsite power production followed by ice precipitation and power loss and the recovery of the lost power through daylighting and the removal of the ice through deicing (see Video S1, Supporting Information). The fan was operated to generate power onsite through the TPV cell; this included an electric running load of 3.5 mW and a starting load of 6.6 mW. On a snowy day, the ice precipitates resulted in ice sheets of ≈20 mm depth, hampering the functions of the device such as power generation and solar transmittance and its role in maintaining the efficiency of the building and providing comfort to the occupants of the building. The FITE‐TH was switched on for rapid deicing; the TH was operated at a bias voltage of 3 V. After 10 min, power recovery was performed, which also cleaned the device and supported the working of the transparent roof. This result suggested that the FITE‐embedded TPV‐TH can enable the use of renewable energy through energy‐efficient building envelopes in freezing climates.

### 2.7 AEW under Varying Temperatures and Light Intensities

**Figure 5:**
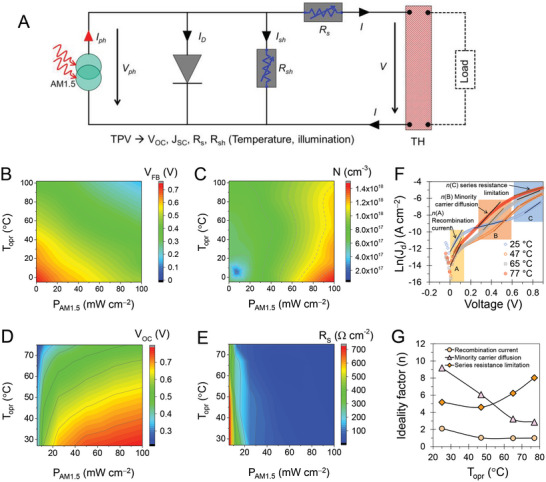
Performance of TPV‐TH under variations in temperature and illumination. A) Equivalent circuits of AEW depict effect of variations in temperature and illumination on performance of TPV‐TH regarding temperature and light intensity functions, B) flat‐band potential (*V*_FB_), C) effective carrier concentration (*N*), D) open‐circuit voltage (*V*_OC_), and E) series resistance (*R*_s_). Diode dark current analysis. F) Ln(*J*_d_) versus V characteristic plots of the TPV‐TH device at various *T*_opr_. G) Summary of diode ideality factor of various current transports.

The AEW operates under conditions that include variations in the temperature and illumination. The TPV and TH units have been integrated in the equivalent circuit to simplify its operation, as shown in **Figure** **5**A. Initially, onsite power production mainly relies on the illumination source, which generates a photocurrent (*I*_ph_) flow against the diode current (*I*_D_) and photovoltage (*V*_ph_) across the TPV. A combination of both of these parameters generated power that was delivered to the load; in the current case, the load was the electric fan, and power was delivered through the TH. The built‐in potential is the main driving force for separating the photogenerated charges, which also originate from the open‐circuit voltage and short‐circuit current. However, under variations in the operational temperature and illumination, the intrinsic parameters of the series resistance (*R*_s_) and shunt resistance (*R*_sh_) regulate the *V*_bi_, *V*_OC_, and FF.

In a typical a‐Si PV cell, the thickness of the absorber layer is 150–300 nm, which contributes to the light‐induced metastable properties in hydrogenated a‐Si (a‐Si:H). During illumination, a degradation effect briefly occurs in the a‐Si:H layer; this effect is characterized by an increase in the unpassivated dangling bonds, which eventually tend to saturate at a higher value after 1000 h of light exposure. This effect causes less degradation in a‐Si PV devices with thicknesses of 100 nm or less.^[^ ^50^, ^51^, ^52^ ^]^ Owing to the p‐i‐n structure, the *V*_OC_ depends less on the thickness of the absorber layer and metastable properties; however, the photocurrent dramatically affects the lower output power of the device owing to the increased dangling bonds that promote the recombination of the photogenerated carriers. The original state of the a‐Si:H can be restored by at elevated temperature condition,^[^ ^52^, ^53^, ^54^ ^]^ which could potentially benefit its use in AEW applications.

Mott–Schottky (M–S) analysis is a powerful method for characterizing TPVDs that have built‐in potential and the effective carrier density to reveal the usefulness of a‐Si devices and their metastable properties.^[^ ^55^, ^56^ ^]^ Figure S17 (Supporting Information) shows the MS linear plots of the capacitance of the space charge region for dark, low‐level injections (20 mW cm^−2^), and high‐level injections (100 mW cm^−2^). Markers have been provided to guide the estimated flat band potential (*V*_FB_) and effective carrier density (*N*), which were obtained from Ref.[^[^ ^57^ ^]^] The parameters *V*_FB_, *N*, and *V*_OC_ had a noticeable influence on the level of injection (dark to 1 sun) and temperature (−5 to 102 °C). Figure 5B displays the *V*_FB_ contour for the temperature of the device and light intensity of the AM1.5 spectrum, revealing that the drift‐dominated performance can be attributed to the p‐i‐n structure with the ultra‐thin absorber layer of a‐Si:H. A *V*_FB_ value of 0.15 V was observed even at 100 mW cm^−2^ and 102 °C. On the other hand, the *N* value increases from 10^17^ to 1.5 × 10^18^ cm^−3^ when the light intensity increases to 100 mW cm^−2^ (Figure 5C), which may be attributed to a light‐induced dangling bond, where photocarrier recombination excites mobile H from Si─H bonds, leaving behind dangling bonds.^[^ ^54^, ^58^ ^]^ Interestingly, when the temperature increased, the N value decreased from 1.5 × 10^18^ to 1.1 × 10^18^ cm^−3^, indicating the restoration of the original state by annealing.^[^ ^53^ ^]^ We confirmed that the TH‐embedded TPV–device based on a‐Si, which recovered the original states of a‐Si:H, provided a stable performance.

Furthermore, the temperature and light intensity conditions affected the series resistance (*R*_s_); an increase in temperature and light intensity produced a decrease in *R*_s_. However, owing to the dominant effect of the voltage drop through *R*_s_ due to the increased temperature and reduced light intensity, the *V*_OC_ of the device was lower. The contour plots of *V*_OC_ and *R*_s_ in Figure 5D,E confirm these trends. This result showed that an increased *R*_s_ value under a higher temperature and lower light intensity significantly reduced the *V*_OC_ of the device and hampered onsite production. The *J*_SC_, FF, and *R*_sh_ parameters are shown in Figure S18 (Supporting Information). The PCE of the TPV‐TH depended on the temperature and light intensity. The TPVD provided consistent onsite power in the temperature range up to 50 °C and started to decrease with the increase in temperature for the 1‐sun condition; however, the PCE rapidly decreased at a light intensity below 50 mW cm^−2^, which was attributed to the light‐intensity and temperature‐dependent parasitic losses (FF, *R*_s_, and *R*_sh_). Noteworthily, the onsite power generation of the a‐Si TPV with the FITE design largely depended on the light intensity.

Indeed, dark currents provide valuable insight into various current flow mechanisms. According to the standard diode model, dark *I–V* characteristics dictate specific current flow, i) due to the recombination process within the junction (at lower bias <0.1 V), ii) due to minority carrier diffusion (≈0.4–0.6 V due to lowering of the potential barrier), and iii) current flow limited by series resistance (at higher bias >0.8 V).^[^ ^35^ ^]^ Figure 5F shows temperature dependent Ln(*J*_d_) versus V characteristic plots of the TPV device under dark. The current is systematically varied in the temperature range of 25–77 °C, corresponding to the aforementioned specific carrier processes. Based on the diode ideality factor, the quality of diode can be derived by minority diffusion and corresponding recombination processes in the space charge region. The ideality factor (*n*) can be estimated below by the diode dark current relation.

(5) $$n=\left(\frac{q}{kT}\right)\left(\frac{dV}{d\left(Ln\left(J_{d}\right)\right)}\right)$$

Where kT/q is the thermal voltage, and dV/d(Ln(*J*_d_)) is the slope (as marked in Figure 5F). Table S2 (Supporting Information) summarized parameters corresponding to dark current analysis, revealing the stages of recombination current, minority carrier diffusion, and series resistance limitation. Figure 5G shows the *n* of the TPV device as a function of the operation temperature (*T*_opr_) of the device. Indeed, it clearly provides the reduction of recombination current of TPV devices at the elevated temperature condition (50–77 °C) from the case of 25 °C.

### 2.8 Aesthetics Assessment: Angle‐Dependent Transmittance and Colors

**Figure 6:**
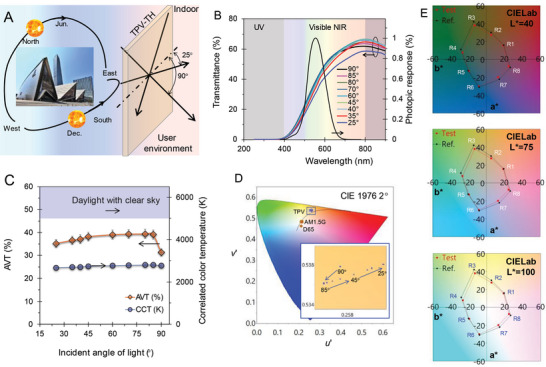
Dependence of aesthetics of AEW on incident angle of light. A) Schematic illustrating influential parameters on aesthetics, including daily/seasonal light variations and users’ position concerning the AEW in the building. B) Transmittance profiles of device position concerning the incident angle of light and the photopic response of the human eye. C) AVT and correlated color temperature (CCT) as a function of the angle of incidence. D) Standard CIE 1976 chromaticity diagram showing color coordinates of the TPV‐TH device at various angular positions (inset shows CIELUV color coordinate of the device). E) CIELab plot of active energy window for L* value of 40, 75, and 100.

The figures of merit for the aesthetic assessment included average visible transmittance (AVT), color coordinates, correlated color temperature (CCT), and color coordinates (CIELab).^[^ ^12^, ^16^, ^40^ ^]^ These parameters have been reported for incident light angles that are normal to the device. The schematic representation in **Figure** **6**A shows the light source (daily and seasonal) and user position, considering the TPVD‐applied structure. Perception through the AEW was governed by our visual angle to the light source, device, and object surface, creating the need for angle‐dependent aesthetic assessments. In this context, the angle‐dependent transmittance of the AEW on the fixed optical path helped to estimate the AVT, color coordinates, and correlated temperature. It can support standards that benefit the aesthetic parameters of AEW. Figure 6B shows the transmittance profiles for angles of 25°–90°, with the baseline of the AEW, a blank measurement in the air. The consistent transmittance over the full spectrum for wide angular positions is attributed to thin‐film structure and compact electrodes. Using the following relation, we estimated the AVT in the wavelength region of 380–780 nm for the angular positions:^[^ ^14^ ^]^

(6) $$AVT\left(\%\right)=\frac{\int_{380\;\mathrm{nm}}^{780\;\mathrm{nm}}T\left(\lambda \right)P\left(\lambda \right)S\left(\lambda \right)d\lambda }{\int_{380\;\mathrm{nm}}^{780\;\mathrm{nm}}P\left(\lambda \right)S\left(\lambda \right)d\lambda }$$

where *T*, *P*, *S*, and *λ* are the transmittance, photopic response, solar photon flux (AM1.5G), and wavelength, respectively. The AVT values of the angularly positioned device are summarized in Figure 6C; there is an abrupt rise at an angle of incidence of 85° that shows a consistency of 39 ± 1% over the angle from 45° to 85°; this consistency then slightly reduces to 35% at 20°, demonstrating visual light consistency. Further, it is worth noting that AEW provides excellent UV blocking (100%) and daylighting of 196 W m^−2^, as revealed from the transmittance and absorbance profiles for various incident angle of light (Figure 6B; Figure S19, Supporting Information).

The International Commission on Illumination (CIE)−1976 standard can be adopted for angular color assessment to project color coordinates (*u’*, *v’*); the method used to calculate (*u’*, *v’*) is given in Section S1 (Supporting Information), color analysis. The CIE 1976, also called CIELUV, was used to provide a more uniform color spacing than CIE 1931 did for colors with approximately the same luminance.^[^ ^59^ ^]^ The estimate (*u’*, *v’*) of the device for various angular positions was obtained using a chromaticity diagram obtained by employing ColorCalculator v7.77; this diagram is shown in Figure 6D. The CIELUV color coordinates of the AEW were (0.257, 0.538) for the 90° incident light angles; decent color homogeneity was observed over the angular positions 90°–25°, as seen in the inset in Figure 6D.

The CCT measures the color appearance of a light source, defined by the closeness in distance between the chromaticity coordinates and blackbody locus.^[^ ^60^ ^]^ The CIELUV can help estimate the CCT value of the device, as described in Section S1 (Supporting Information). The CCT value of the device with the angle of light incidence revealed a range of 2700–2800 K, as shown in Figure 6C (secondary axis). The angular aesthetic assessment parameters are summarized in Table S3 (Supporting Information).

Further, colors of various objects can be quantitatively examined by the color rendering index (CRI) in comparison with a standard light source. To calculate the CRI value of AEW, we used the free format spectrum of the input spectrum as a function of photon flux. The photon flux spectrum of the standard light sources shows the color appearance of AEW device, to obtain the CRI values (Figure S21 and Table S4, Supporting Information). This result validated the CRI value of 100 for the reconstituted daylight and 98 for blackbody spectrum of 5800 K, respectively. This result confirmed the CRI value of 92 for the AEW for 90° of the incidental angle of light. These high CRI values of AEW indicate the outstanding performance, compared to the standard requirement CRI value (>70) or excellent grade (>85).^[^ ^14^ ^]^ Moreover, the AEW performance confirmed the unique CRI values despite of varying the incident light angles. Angle‐dependent transparency and color appearance are the critical factor of TPV deployment for residents. The wide‐angle of view should be guaranteed to realize the TPV power generation on the window of buildings^[^ ^61^ ^]^ or human interface devices.^[^ ^62^ ^]^

The CIELab color space can quantitatively derive color perception through a window, also referred to as L*a*b*, defined by the CIE. In this, L* is for perceptual lightness, and a* and b* are for the four unique colors of human vision: red, green, blue, and yellow. It was designed to confirm color identification of a perceived color configuration.^[^ ^14^, ^40^ ^]^ The CIELab value of AEW and the corresponding colors of the test samples (R1–R8) are shown in Figure 6E. The CIELab parameters of AEW samples presented the identical color performances under the reconstituted daylight of 5800 K and blackbody irradiance of 5800 K (Figure S22 and Table S5, Supporting Information). From the CIELab parameters, it is possible to extract the total color difference (ΔE*). It is estimated that ΔE* values from 1to 6 are considered to be good color configurations. The values between 6 and 9 are considered susceptible to visual sensation; meanwhile, it is considered as low quality above 10. It is worth mentioning that the AEW samples possess ΔE* values in the range of 1–6 for R1–R8 colors and show consistent color appearances.

The results of the angular AVT, CIELUV, CCT, and CIELab demonstrated aesthetic assessment, suggesting the suitability of the AEW with the FITE‐embedded TPV‐TH for incorporation into pleasant building envelopes in freezing climates to adopt renewable energy. The integrated device of the TPV‐TH is based on the ultrathin a‐Si absorber as a light harvester and FITE as the TE serves as a device that provides onsite power, thermal‐visual comfort, daylighting, and weatherization for a clean energy system.

## 3 Conclusion

In summary, a strategy for abundant sunlight deployment through buildings using an AEW that integrates a FITE‐embedded TPV‐TH device is presented. Onsite energy production, daylighting, UV blocking, and deicing were demonstrated using a simple, durable, scalable, inorganic‐thin‐film‐based device. The transparent conductor of the AZO/AgNW/ZnO nanostructure played a vital role in developing a high‐performance device through field‐induced tunnel electron transport, offering a PCE of 3.07%, an AVT value of 43.2%, and durable operation. We tested the thermal durability of the device under extreme conditions such as high temperatures such as 300 °C and ice covers using TH‐TPVs with a state‐of‐the‐art setup under a standard solar simulator; we also set up a benchmark to test transparent PV technology. The integrated system provided onsite power generation with a power density of 30.7 W m^−2^, high transmittance, and angular aesthetics, thereby significantly reducing the energy consumption of buildings for electricity, lighting, and thermal regulation. The developed system also provided a see‐through color perspective, perfect UV shielding, daylighting, thermal comfort, and a deicing function for the building envelope. This system can be a promising energy flow entity owing to its widespread solar utilization capabilities, even under extreme heat and snowy conditions.

## 4 Experimental Section

### Device Fabrication

Fluorine‐doped tin oxide (FTO)‐coated glass (735 159 Aldrich, sheet resistance 7 Ω cm^−2^) was used as the conducting substrate. The substrates were cleaned in acetone, methanol, and deionized water using an ultrasonic cleaner (15 min each) and then dried under nitrogen stream.

A plasma‐enhanced chemical vapor deposition (PECVD) system was used for the a‐Si process. Layers of 25, 20, and 25 nm, respectively, of p‐, i‐, and n‐type a‐Si were directly deposited on the FTO glass. A radio‐frequency (RF) power source (13.56 MHz, 56 mW cm^−2^) was used to ensure the glow discharge decomposition of the silane (SiH_4_) and hydrogen (H_2_) gas mixtures. Diborane (B_2_H_6_) gas was used for the p‐Si, and phosphine (PH_3_) gas was used for the n‐Si.^[^ ^63^ ^]^ An AZO layer was deposited on top of the n‐Si layer by magnetron sputtering (SNTEK, South Korea). The AZO target (iTASCO, purity 99.99%) was sputtered with Ar at a gas flow rate of 5 sccm at 200 °C using an RF power of 200 W for 15 min. For the top electrode and TH assembly, AgNW ink (∅20 nm, 20 µm length, Flexiowire, South Korea) was spin‐coated at a rotational speed of 2000 rpm, and 70 µL of ink was dispensed using a micropipette to prepare the AgNW film. Thermal treatment at 150 °C for 1 min was performed by rapid thermal processing to form the AgNW network. Magnetron sputtering was used to deposit the top ZnO film (ZnO target, iTASCO, purity 99.99%). Ar with a gas flow rate of 50 sccm was used to prepare the hybrid ZnO/AgNW samples with an RF power of 50 W and process pressure of 5 mTorr. Silver paste (ELCOAT P‐100) was applied to the ZnO layer for producing the electrodes of the THs. Rapid thermal processing was applied to study the structural stability of the AgNW and AgNW/ZnO electrodes. A TH and hot plate were used as the thermal devices for the ambient studies.

### Characterization

The optical properties, including the transmittance, absorbance, and reflectance, of the samples were measured using a UV–vis–NIR spectrophotometer (Shimadzu, UV‐2600) equipped with a diffused integrated sphere. The baseline was a blank measurement in air; the measurement range of the optical wavelength was 280–1400 nm, and the step wavelength was 5 nm. Angle‐dependent optical measurements were performed without a diffused integrated sphere directly attached to the angular control of the device on the optical path.

The sheet resistance and carrier mobility were measured using a Hall measurement system (AHT5573R, Ecopia, Anyang, South Korea); the thickness parameters for these Hall measurements were obtained using a spectroscopic ellipsometer (J.A. Woolam, α‐SE). A magnetic field of 0.55 T and compliance current were applied throughout the Hall measurements. The silver paste was used for creating metal contact with the contacts of a van‐der‐Pauw device and then dried for 2 h. Calibration was achieved using standard ITO and GaN samples to verify the reliable mobility and sheet resistance.

The structural properties of the AgNWs and ZnO/AgNWs were obtained using XRD (Rigaku, SmartLab) with Cu K‐α radiation (*λ*_Kα_ = 1.5405 Å). A grazing mode with a glancing angle of 1°, X‐ray power of 45 kV, current of 200 mA, and incident slit of 1 mm was used with steps of 0.02° (2θ). The surface morphologies were analyzed using field‐emission scanning electron microscopy (FESEM; JEOL, JSM‐7001F).

The structural, physical, and elemental properties of the AgNW/ZnO hybrid were analyzed using field‐emission transmission electron microscopy (FETEM, JEOL, JEM‐2100F). TEM specimens were prepared directly on a TEM grid (300 mesh, 3.0 mm O.D., Copper, PELCO, 1GC300). A focused ion beam was used to prepare the specimen of the device and obtain the cross‐section and elemental mapping. The temperature of the TH system was obtained using a real‐time module developed using an Arduino Uno. A K‐type thermocouple was used as the temperature sensor and attached to the device with proper electrical insulation.

The current‐voltage characteristics of the TH and TPVD were measured using a source measure unit (SMU, Keithley 2400, Keithley Instruments, Beaverton, OR, USA) and a potentiostat/galvanostat (PGSTAT, ZIVE SP2, WonATech, Seoul, Korea).

A simulator system (McScience‐K300, Korea) and PV power meter (McScience‐K101, Korea) were used to measure the performance of the TPVD. The light intensity was varied from 100 to 10 mW cm^−2^ by controlling the current source to the xenon arc lamp. The quantum efficiency (QE) of the TPVDs was measured using QEX10 (PV Measurements, Inc.). For stability studies, a temperature of up to 300 °C for a ramp of 100 °C min^−1^ and a hold time of 600 s was provided by a hot plate under ambient conditions (60 relative humidity (RH)).

The TH was used to study the temperature‐dependent performance of the TPVDs under a solar simulator, and a real‐time thermocouple module was used to monitor the temperature. All thermal measurements under the solar simulator were performed at a room temperature (RT) of 25 ± 1 °C. All the components of the TPVD were custom‐designed, and measurement set‐ups were developed to enable the see‐through device measurements to meet the highest standards. An ice cube was applied to the TPVD‐TH device under icy weather conditions to study its performance, which included its deicing and onsite power production capabilities; the TPVD‐TH module had been slightly tilted toward the ice‐melted water channel.

## Conflict of Interest

The authors declare no conflict of interest.

## Supporting information

Supporting Information

Supplemental Video 1

## Data Availability

The data that support the findings of this study are available in the supplementary material of this article.
