# Supplementary material for: Field‐Induced Transparent Electrode‐Integrated Transparent Solar Cells and Heater for Active Energy Windows: Broadband Energy Harvester
Source: Adv Sci (Weinh). 2023 Jul 12;10(26):2303895. doi: 10.1002/advs.202303895 (PMC10502661; doi:10.1002/advs.202303895)
Supplement: Supplementary file 1 — Supporting Information [file ADVS-10-2303895-s002.pdf]

ADVANCED SCIENCE
Open Access

Supporting Information

for *Adv. Sci.*, DOI 10.1002/advs.202303895

Field-Induced Transparent Electrode-Integrated Transparent Solar Cells and Heater for Active Energy Windows: Broadband Energy Harvester

*Malkeshkumar Patel, Sangho Kim and Joondong Kim**

Supporting Information

Field-Induced Transparent Electrode-Integrated Transparent Solar Cells and Heater for Active Energy Windows: Broadband Energy Harvester

*Malkeshkumar Patel, Sangho Kim, and Joondong Kim**

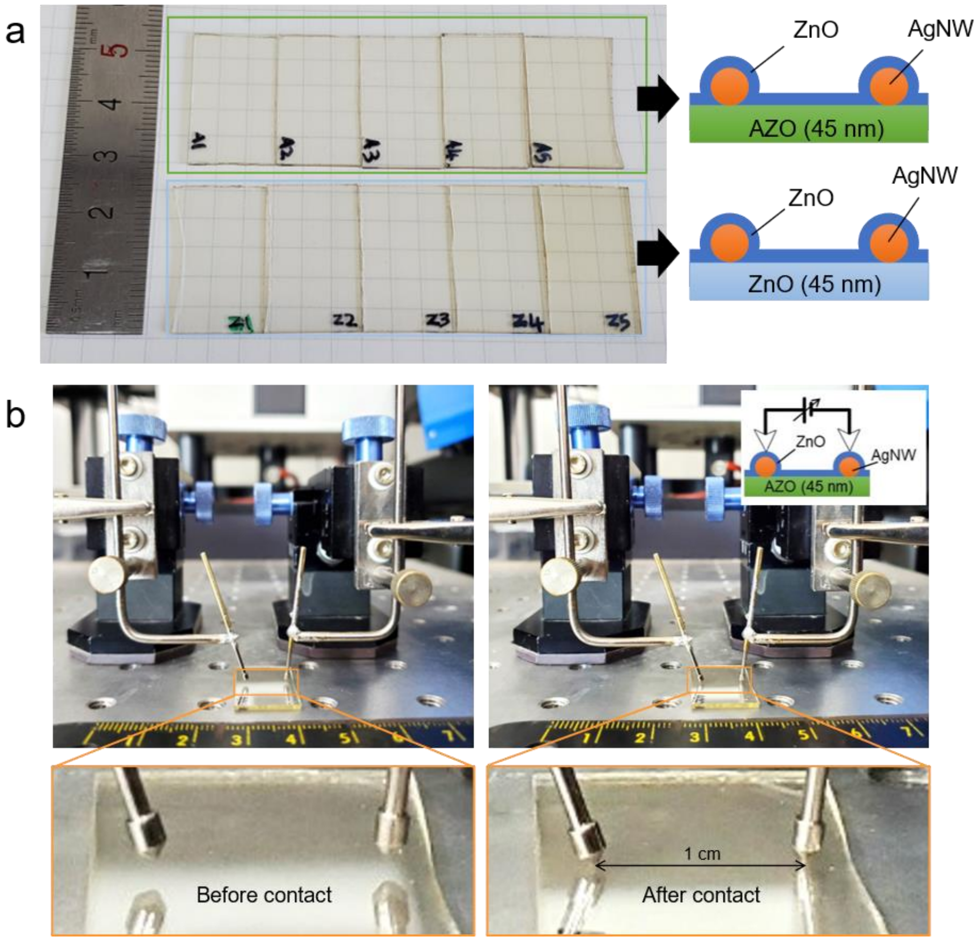

Figure S1. (a) Transparent electrode samples. (b) Setup of current measurement using linear sweep voltammetry.

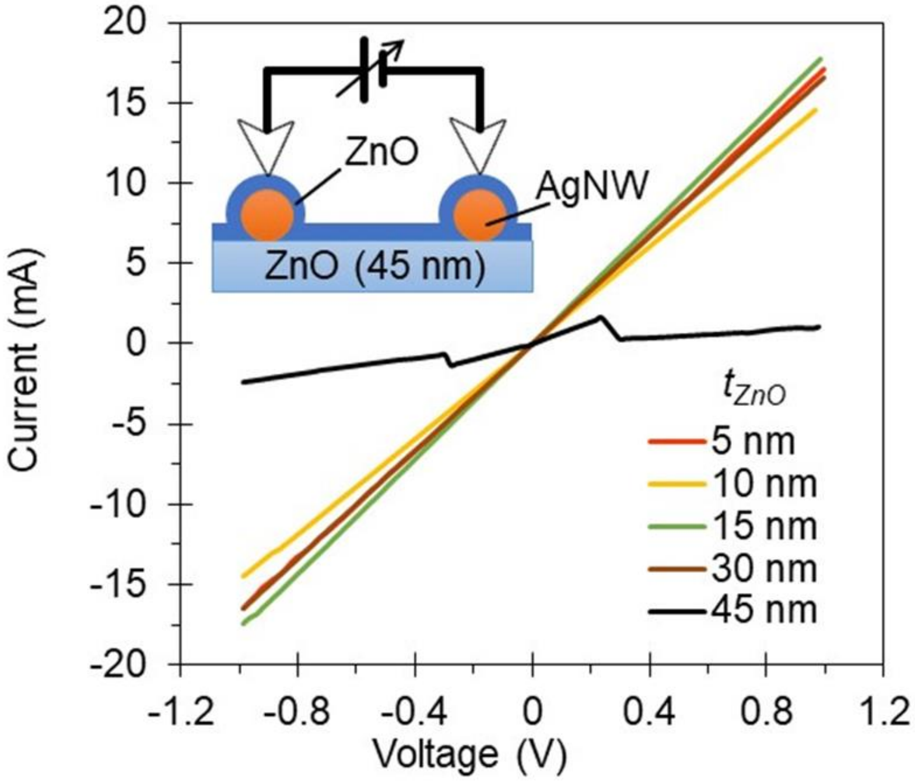

Figure S2. Current-voltage characteristics of TEs (ZnO/AgNW/ZnO) with top ZnO having thickness from 5 to 45 nm (inset schematic shows measurement setup with an electrode spacing of 1 cm).

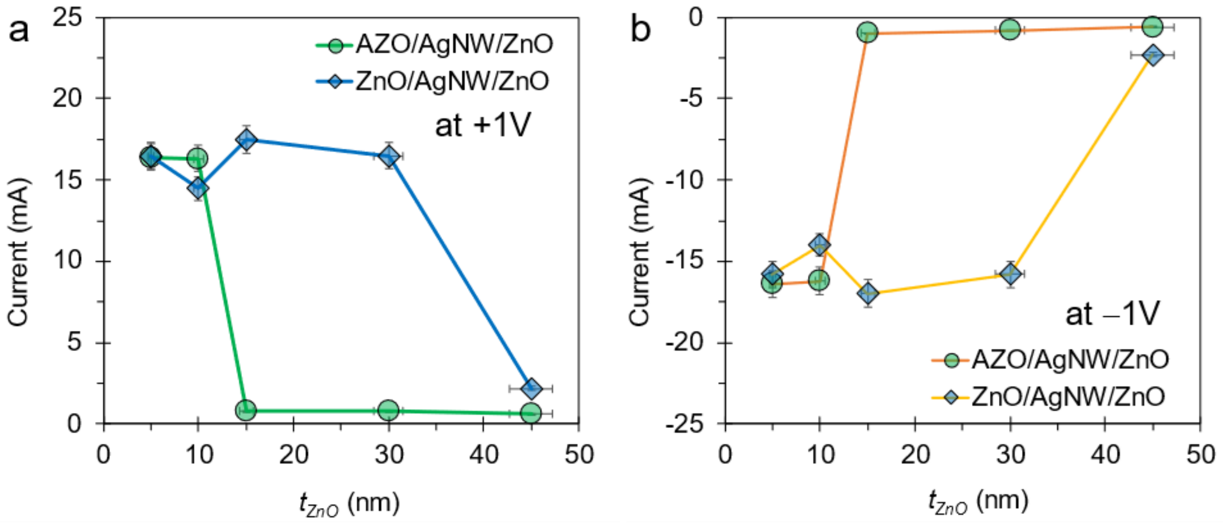

Figure S3. The measured current of AZO/AgNW/ZnO and ZnO/AgNW/ZnO electrodes as a function of top ZnO layer thickness (t_{ZnO}). (a) At +1 V and (b) at –1V.

Table S1. Summary of current measured at +1V and –1V of bias for AZO/AgNW/ZnO and ZnO/AgNW/ZnO electrodes.

t_{ZnO} (nm)	Current (mA) at 1V		Resistance (Ω) at 1 V		Current (mA) at -1 V		Resistance (Ω) at -1V	
	**AZO**	**ZnO**	**AZO/AgNW/ZnO**	**ZnO/AgNW/ZnO**	**AZO**	**ZnO**	**AZO/AgNW/ZnO**	**ZnO/AgNW/ZnO**
5	16.4	16.5	60.98	60.61	-16.4	-15.8	60.98	63.29
10	16.3	14.5	61.35	68.97	-16.2	-14	61.73	71.43
15	0.8	17.5	1250.00	57.14	-1	-17	1000.00	58.82
30	0.8	16.5	1250.00	60.61	-0.8	-15.8	1250.00	63.29
45	0.6	2.2	1666.67	454.55	-0.6	-2.3	1666.67	434.78

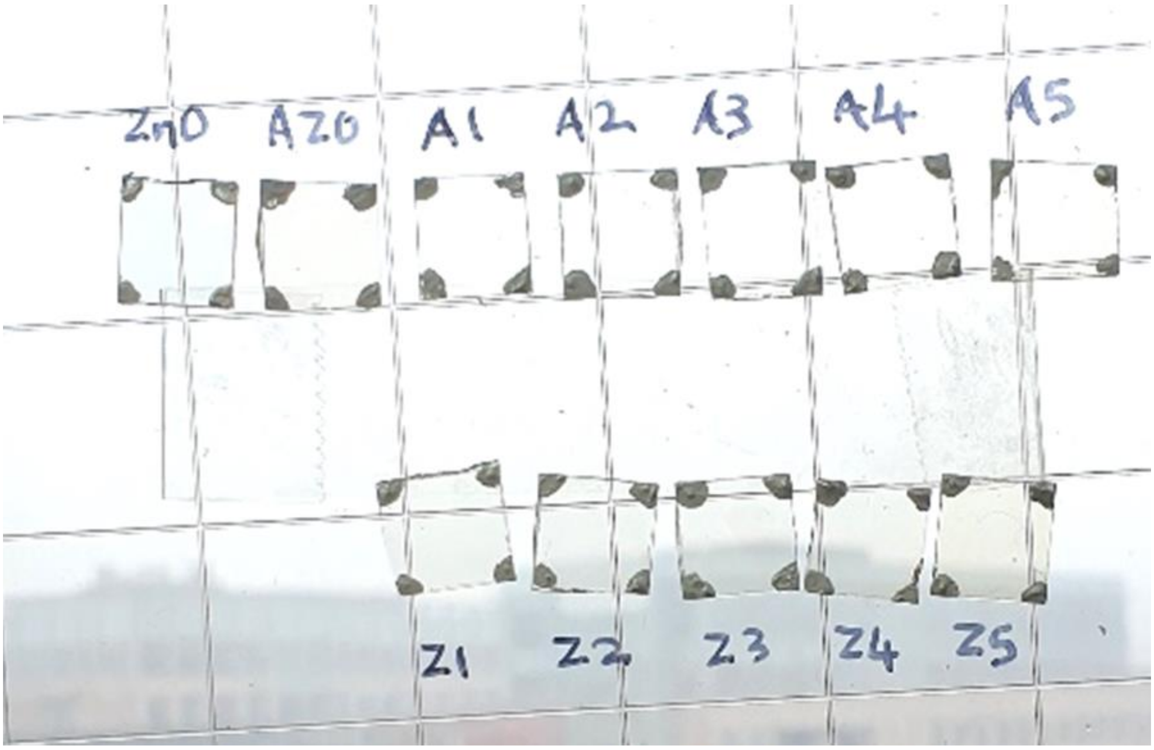

Figure S4. Photocopy of samples prepared for Hall measurement studies. The thicknesses of the ZnO and AZO layers have been calibrated by ellipsometry. The sample size is 1 cm^2, and Ag paste has been applied for metal contact. Samples A1–A5 denote the ZnO/AgNW/AZO configurations, and samples Z1–Z5 denote the ZnO/AgNW/ZnO configurations. The thickness of the top ZnO layer varies in steps of five: 5, 10, 15, 30, and 45 nm.

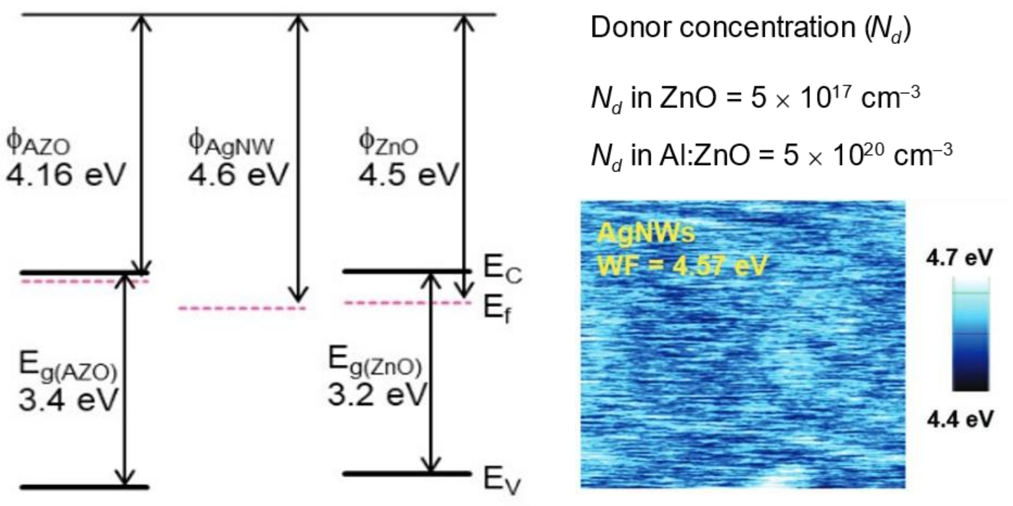

Figure S5. Energy levels and electrical properties of AZO, AgNW, and ZnO. Donor concentration (N_d) in AZO films was 5 × 10^{20} cm^{-3} as confirmed by Hall measurement.[S1] While the N_d in ZnO film was 5 × 10^{17} cm^{-3} as confirmed by Mott-Schottky analysis.[S2] Furthermore, we confirmed the work function of AgNW of 4.6 eV using kelvin probe force microscopy.[S3]

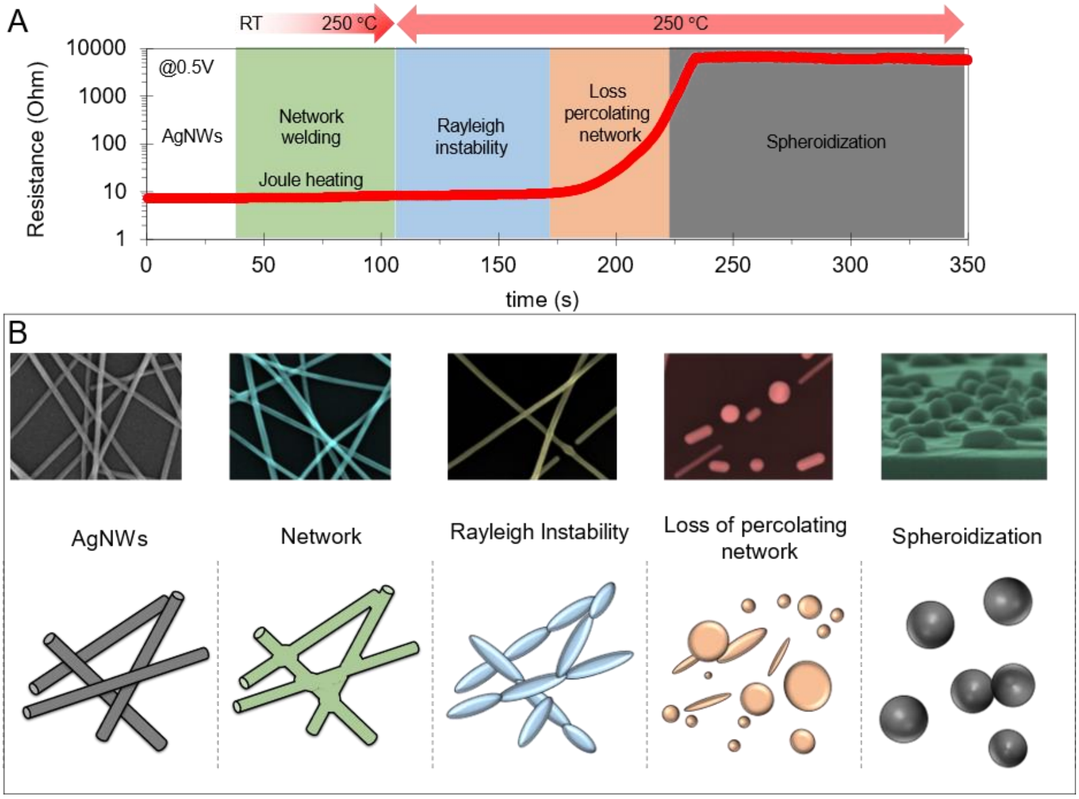

Figure S6. Transient thermal measurement for bare AgNW specimen. (a) Resistance trajectory at 0.5 V of bias during the thermal cycle. (b) Topography of AgNW specimens at a specific interval of the thermal cycle illustrates various phases of networking, Rayleigh instability, loss of percolating network, and spheroidization.

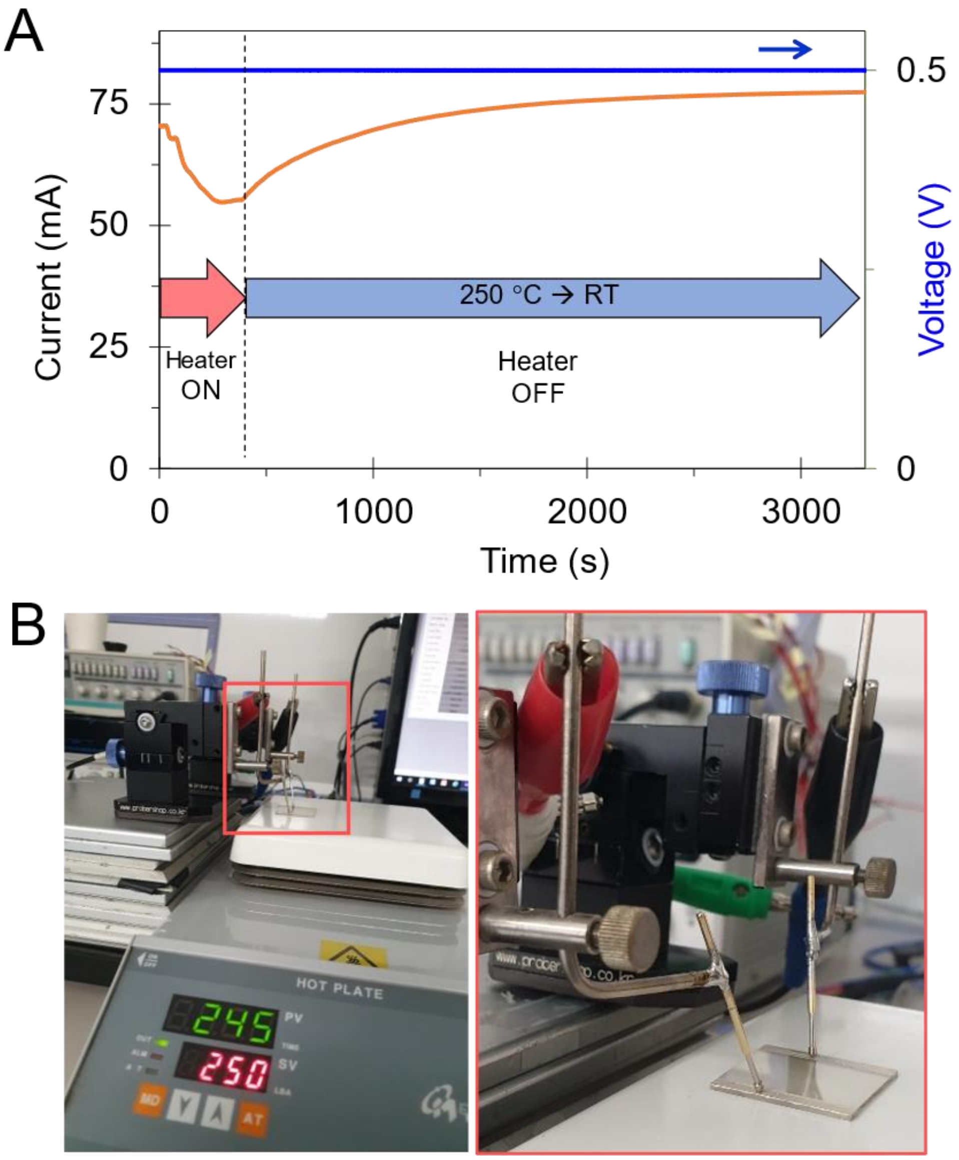

Figure S7. (A) Electric conductance through FITE-based AZO/AgNW/ZnO sample under thermal heating and cooling at 0.5 V bias. (B) Photocopy of thermal heating and cooling of AZO/AgNW/ZnO sample on the hotplate.

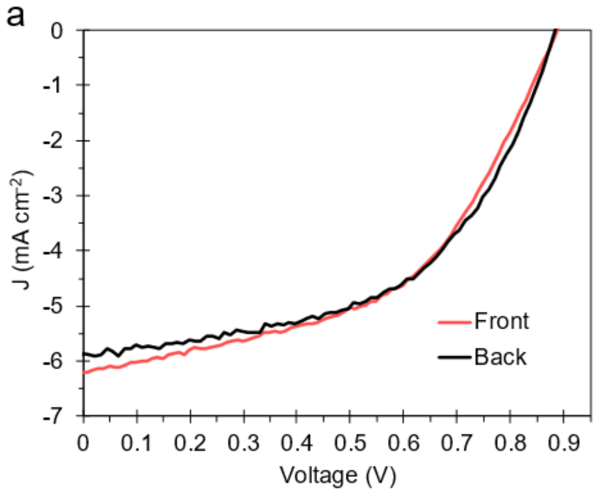

b

Parameters	AZO-AgNW-ZnO	
	Front	Back
Area (cm^2)	3	
AM1.5G intensity (mW cm^{-2})	100	
Temperature (K)	300	
V_{OC} (mV)	890.2	888.26
J_{SC} (mA cm^{-2})	6.21	5.91
FF (%)	55.56	56.55
PCE	3.07	2.97
Bifacial ratio (PCE_{front}/PCE_{back})	1.0342	

Figure S8. Bifacial performance of FITE embedded TPV device. (a) Current density-voltage (J-V) characteristic plots of devices with ZnO/AgNW electrodes under the standard solar simulator (AM1.5G, 100 mW cm^{-2}). (b) Photovoltaic performance parameters for fron and back incident sunlight.

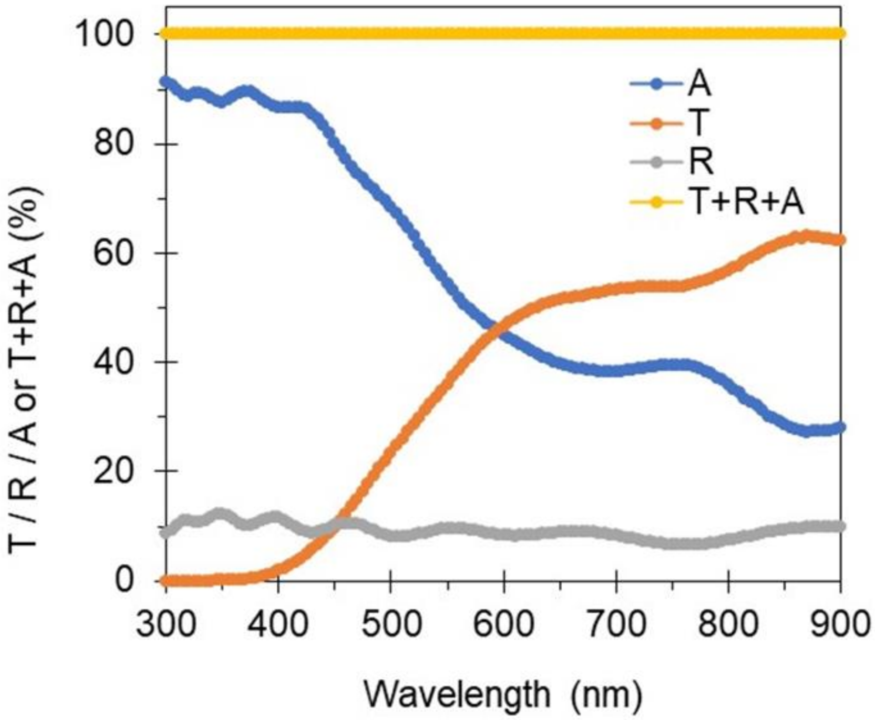

Figure S9. Optical profiles of the TPV device (TPVD) with the ZnO/AgNW/AZO top electrode. Here, A, T, and R are the absorption, transmittance, and reflectance, respectively. The absorption can be derived from the measured R and T value using the relation A = 100 – (T + R), where T+R+A = 100%.

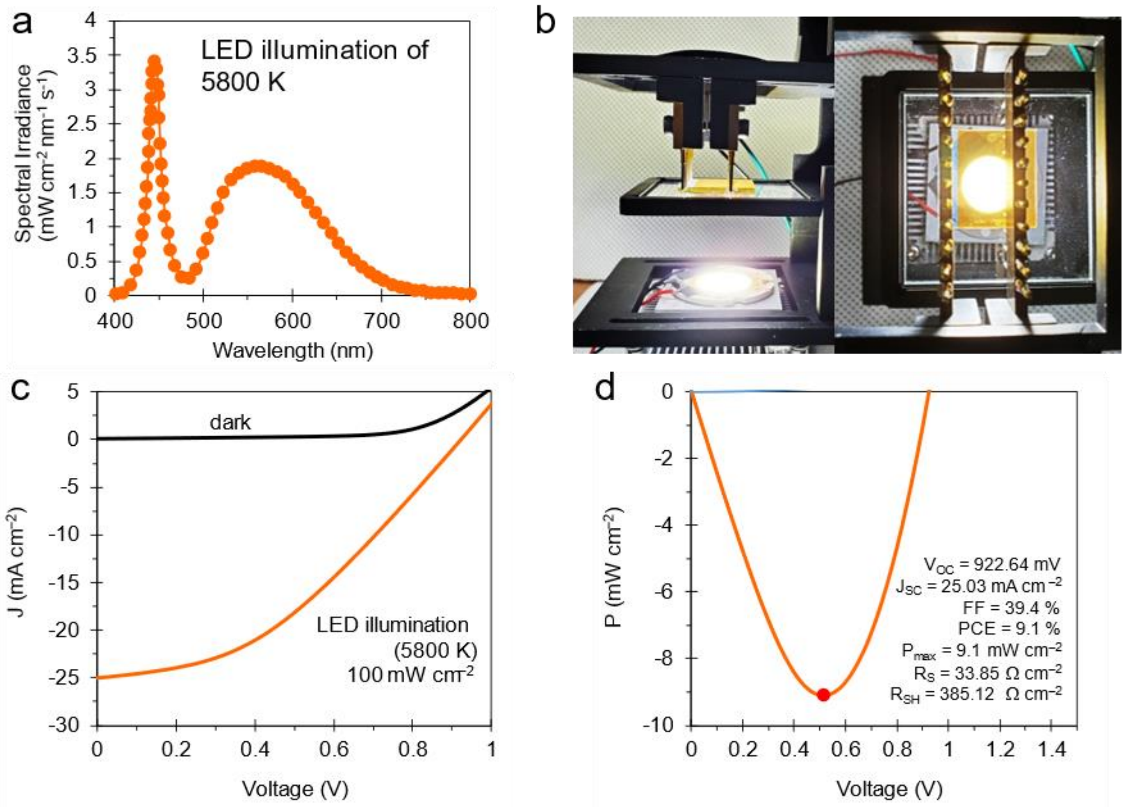

Figure S10. Indoor power production of TPV. (a) Spectral irradiance of LED illumination of 5800 K with a total power density of 100 mW cm^{-2}. (b) TPV-device under the test setup of indoor LED illumination. (c) Current density-voltage (J-V) characteristic plots of the TPV device with ZnO/AgNW/AZO FITE electrode. (d) Power density-voltage (P-V) characteristic plot and photovoltaic performance parameter of the TPV device under LED illumination of 100 mW cm^{-2}.

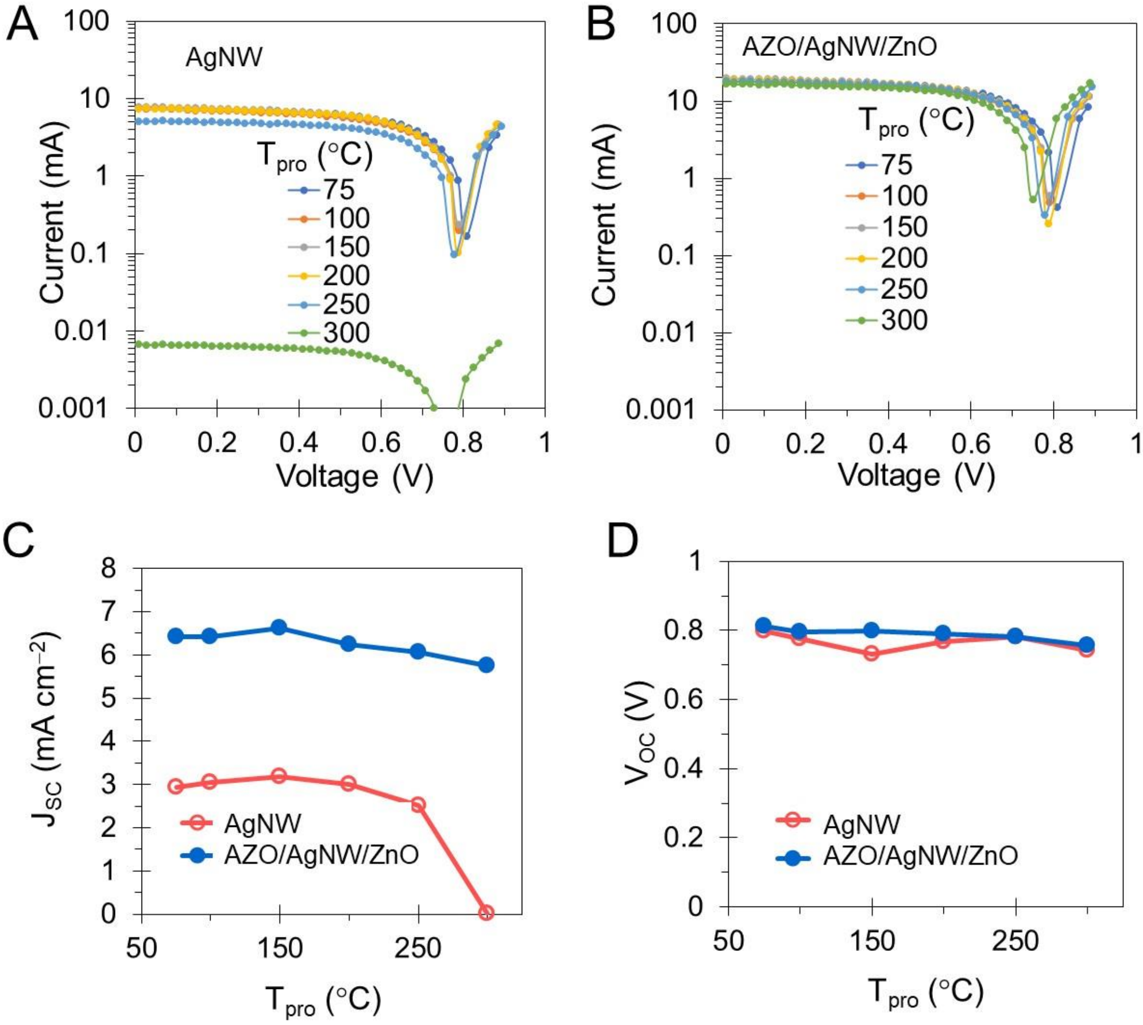

Figure S11. Performances of TPVDs with AgNW and AZO/AgNW/ZnO electrodes under the standard solar simulator. Before measurements, TPVDs are applied at various temperatures from 75 to 300 °C in the air, followed by natural cooling. (A) I-V characteristic plots of a TPV device with AgNW electrode. (B) I-V characteristic plots of a TPVD with AZO/AgNW/ZnO electrode. (C) Short-circuit current density (J_{SC}) and (D) open-circuit voltage (V_{OC}) as a function of processing temperature (T_{pro}) of TPV with AgNW and AZO/AgNW/ZnO electrodes.

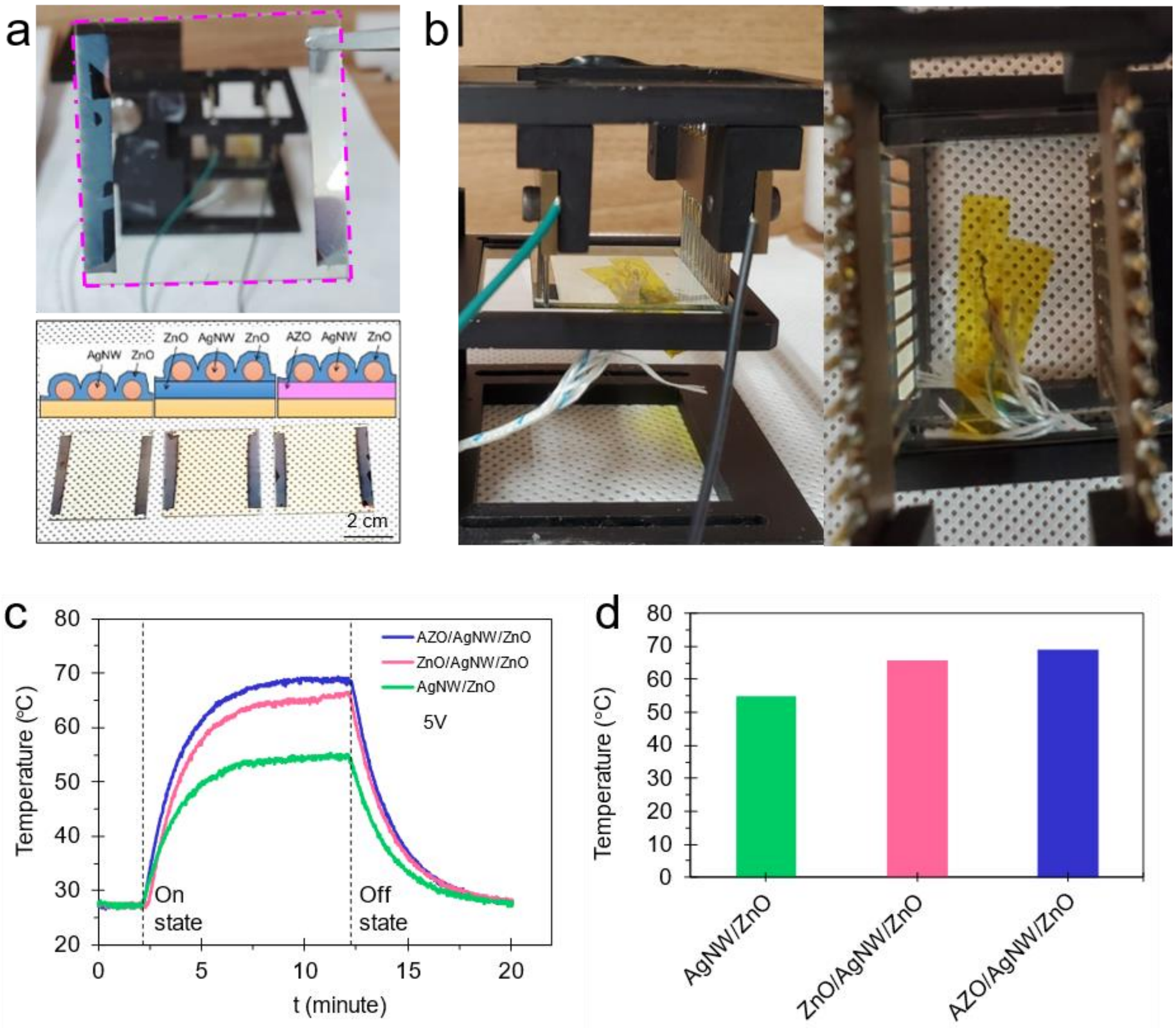

Figure S12. Evolution of the Joule heating performance of the TH. (a) Large area and see-through TH of AgNW/ZnO, ZnO/AgNW/ZnO, and AZO/AgNW/ZnO. The thickness of the top ZnO layer was 15 nm in all heater structures, while the bottom layer of ZnO and AZO film has a thickness of 30 nm. (b) The state-of-the-art TH measurement setup. (c) Transient temperature profiles of the heaters for a total cycle time of 20 minutes. 5V of bias was applied during the on-state. (d) Summary of temperature value of various TH.

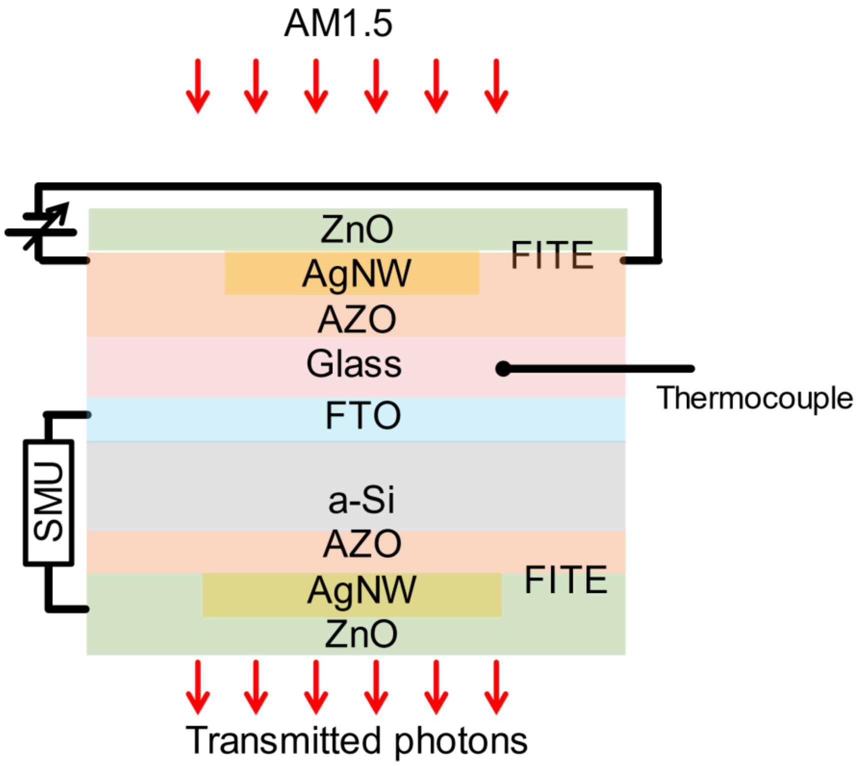

Figure S13. Schematic presenting FITE-embedded TPV-TH device measurement where the steady-state temperature of the device is regulated by the TH coupled with a thermocouple feedback unit. The steady-state light intensity of AM1.5 is regulated by the control unit coupled with a solar power meter.

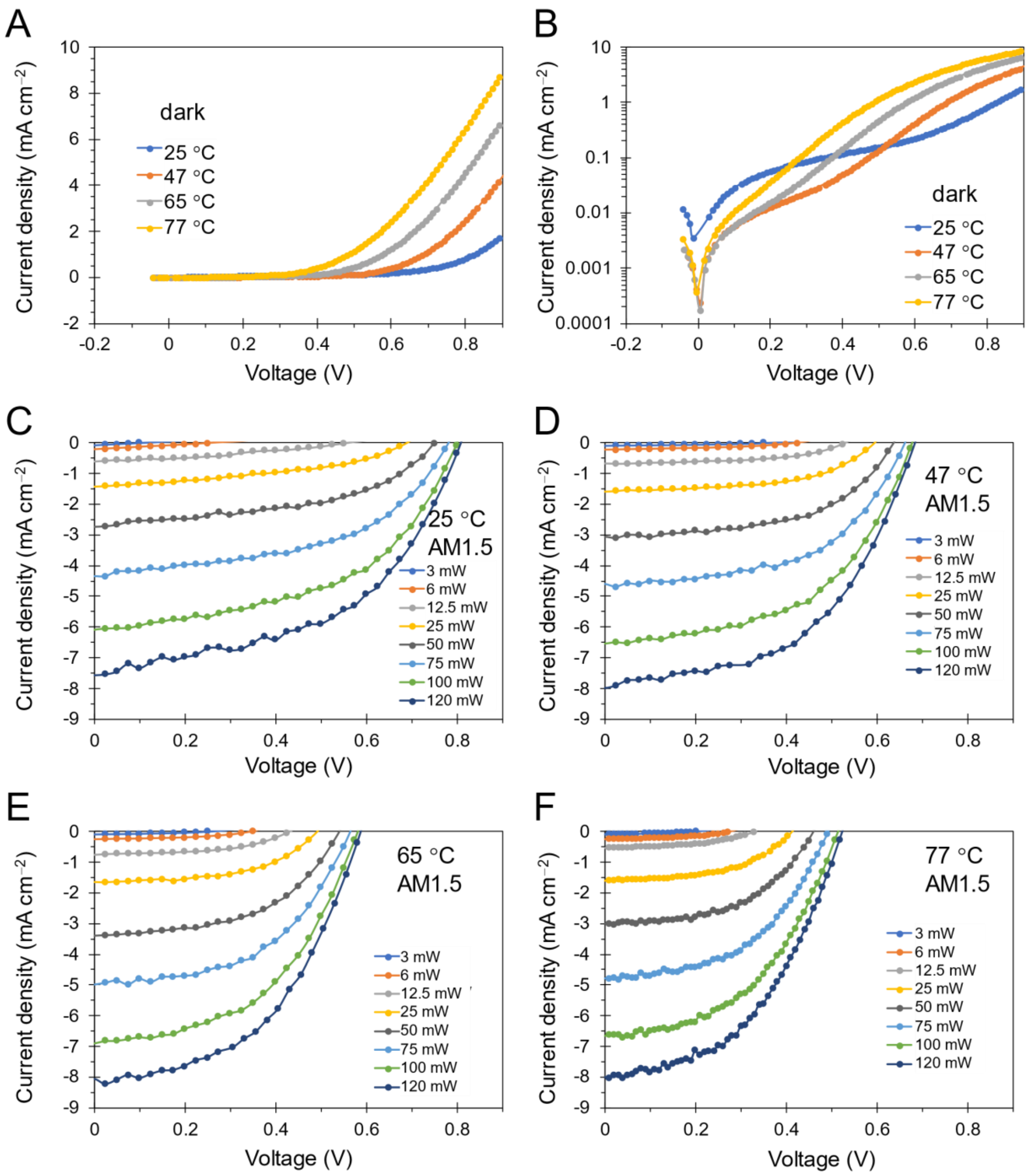

Figure S14. Current density-voltage (J-V) characteristic plots of TPV with AZO/AgNW/ZnO-FITE–top electrode at various temperatures under a dark and varying light intensity of AM1.5. The steady-state temperature of the TPV has been regulated using the integrated TH device and feedback thermocouple module. TPV-TH device under the condition of darkness and increase in temperature from 25 to 77 °C; (A) linear scale and (B) semi-log scale. Device under variation in light intensity from 3 to 120 mW cm^{-2} at (C) 25 °C, (D) 47 °C, (E) 65 °C, and (F) 77 °C.

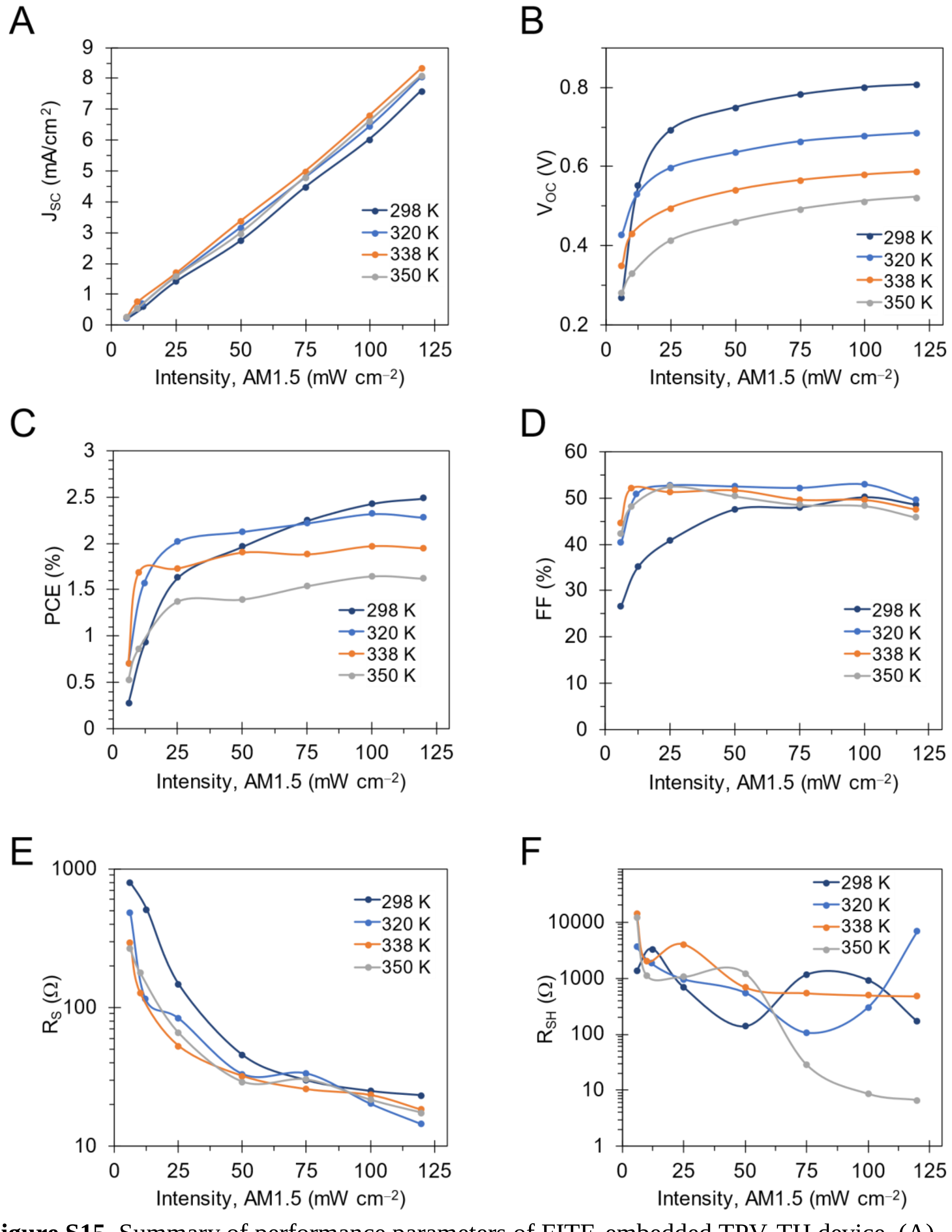

Figure S15. Summary of performance parameters of FITE-embedded TPV-TH device. (A) J_{SC}, (B) V_{OC}, (C) PCE, (D) FF, (E) R_S, and (F) R_{SH} as a function of light intensity of AM1.5.

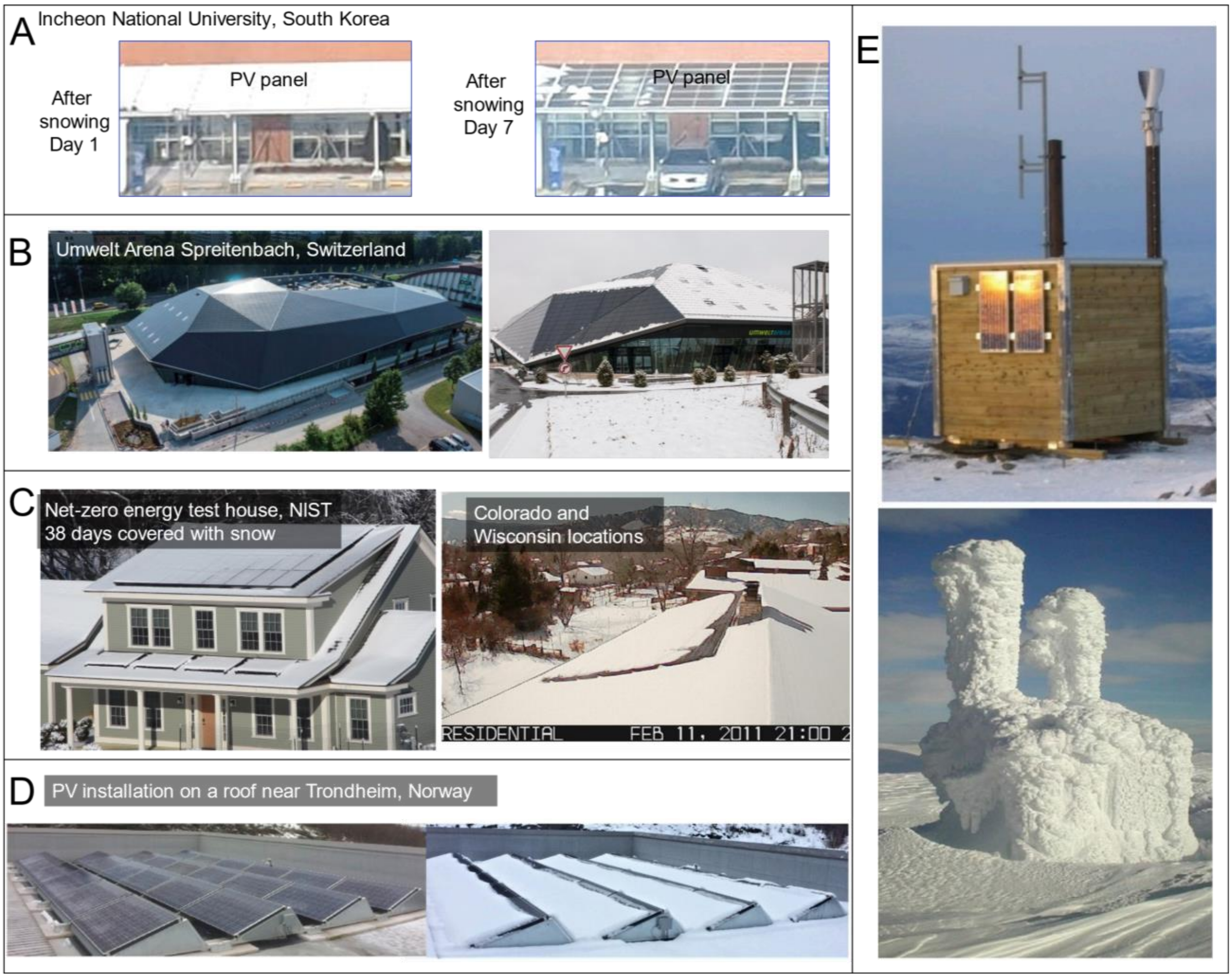

Figure S16. Scenario of photovoltaics (PVs) on a snowy day and under extreme weather at various places. (A) Incheon National University, South Korea, PV panel on a snowy day, and seven days later. (B) Umwelt Arena Spreitenbach, Switzerland, building with energy greater than 203% having customized 750 kWp full-roof building-integrated photovoltaic (BIPV) skin.[S4] (C) Net-zero energy test house at the National Institute of Standards and Technology (NIST) in suburban Washington, and 100-kW rack-mounted PV system on a flat roof,[S5] Colorado, United States.[S6] (D) Left: Frost accretion on typical PV installation on a roof nearby. Right; snow-covered PV installation on a roof near Trondheim, Norway. Snow has begun to slide but has been hindered due to fresh build-up.[S7] (E) Extreme ice and snow accumulation on a communications station; before (top) and after (down) accumulation. Image is courtesy of GETEK Energy AS (Norway) [S7]

Note:

1. *PV-energy loss due to snow:*

In snowy and cold locations such as Sweden, North Europe, North America, and Russia, the production of snow-induced PV energy is 100% monthly and 34% annually.

In moderate climates, significant loss levels around 5–6%, with some levels up to 9.3%, have been reported.[S8–S10] Also, PV-energy losses depending on the tilt angle of the PV panel have

been reported. Snow-related energy-losses accounting for 70% during snow season with a tilt angle of 0° and 60% (snow season) with a tilt angle of 35° (Calumet, MI, USA).[S9]

2. Reliability:

Ice–snow accretion on the PV panel and its structure is a significant concern for system reliability in terms of breakage of cells, interconnectors, frames, and glass, and disruption in the structure of the PV module.[S11,S12]

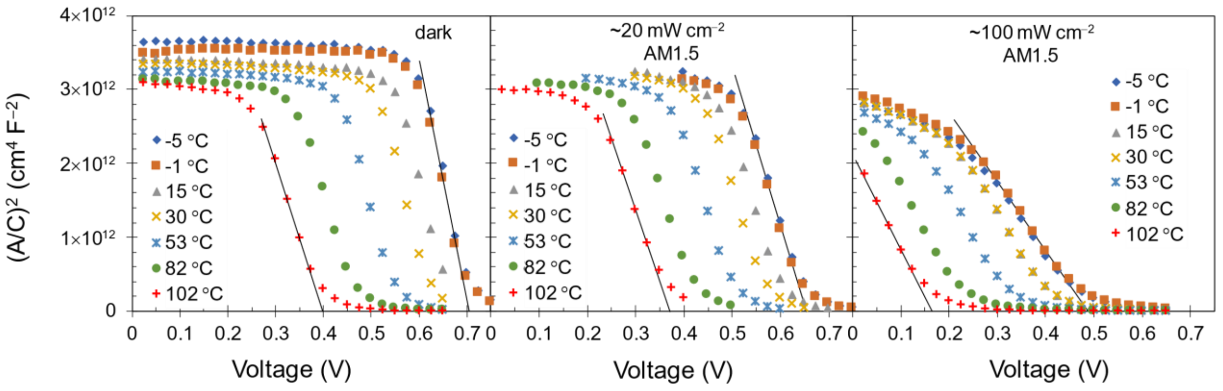

Figure S17. Mott-Schottky (MS) characteristic plots for the TPVD. For each case of illumination, from darkness to 20 mW cm^{-2} to 100 mW cm^{-2} of AM1.5G, the temperature of the device was measured from –5 to 102°C.

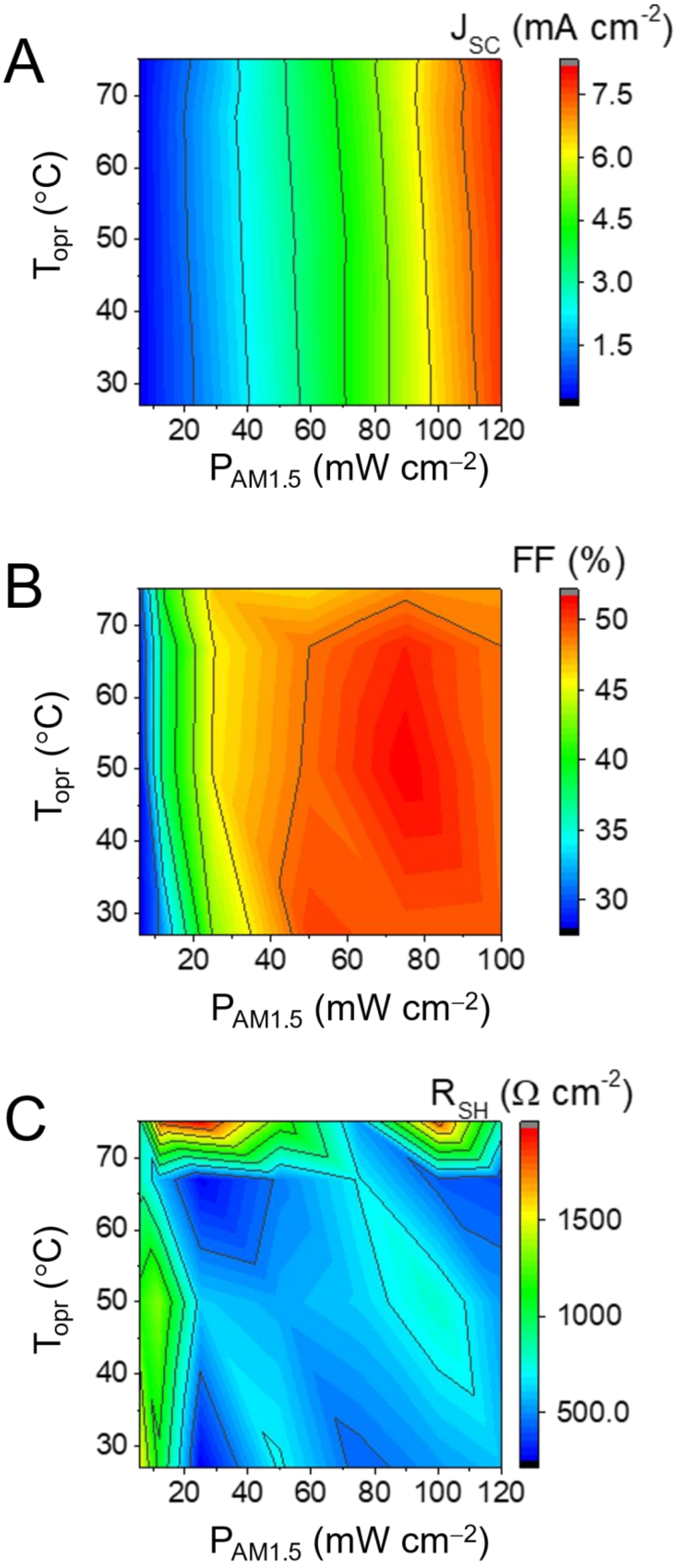

Figure S18. Contour plots of the performance parameters of FITE-embedded TPV-TH device for operational temperature (T_{opr}) and light intensity of AM1.5 ($P_{AM1.5}$). (A) J_{SC}, (B) FF, and (C) R_{SH}.

Table S2. Summary of diode parameters, where kT/q is thermal voltage and *n* is the diode ideality factor.

T_{opr} (°C)	T (K)	kT/q (V)	kT/q (mV)	*Region A Recombination current*		*Region B Minority carrier diffusion*		*Region C Series resistance limitation*	
				d*V*/d(Ln(J_d))	*n*(A)	d*V*/d(Ln(J_d))	*n*(B)	d*V*/d(Ln(J_d))	*n*(C)
25	298	0.0257	25.70	0.0540	2.10	0.2362	9.19	0.1330	5.17
47	320	0.0276	27.60	0.0285	1.03	0.1675	6.06	0.1268	4.59
65	338	0.0291	29.15	0.0287	0.98	0.0942	3.23	0.18165	6.23
77	350	0.0301	30.18	0.0300	0.99	0.0863	2.86	0.2424	8.03

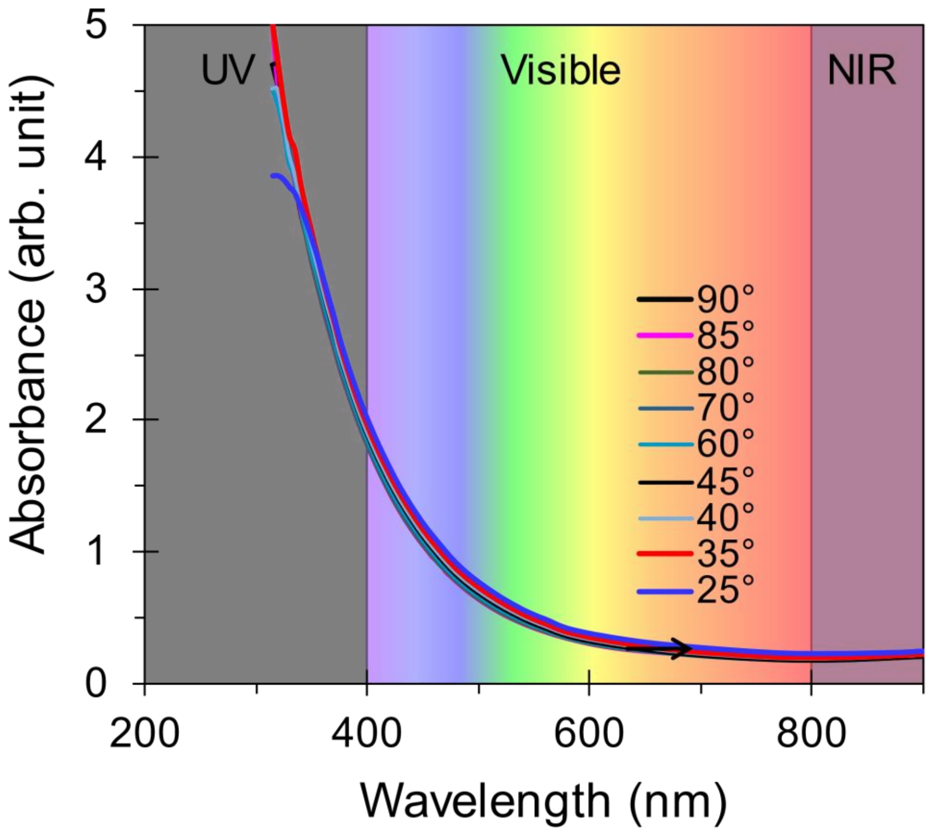

Figure S19. Absorbance profiles of active energy window as a function of photon wavelength. Incident angle of light was varied from 90-25° normal to the AEW.

Supplemental Information Section S1: Summary of color analysis

The figure of merit for color is the color coordinates defined by the International Commission on Illumination (CIE), which quantitatively describes how accurately the color of a given object appears from a light source or through a transparent medium, concerning an “ideal” illumination source (daylight, AM1.5G, and indoor illumination), commonly used in the lighting and window industries.[S13]

We employed the CIE 1976 standard to depict the color perception of the transparent device using XYZ tri-stimulus values. These values can be obtained using the following equation:

$$X = \frac{\int_{380\,nm}^{825\,nm} T(\lambda)P(\lambda)\,x(\lambda)\,d\lambda}{\int_{380\,nm}^{825\,nm} P(\lambda)\,y(\lambda)\,d\lambda} \quad \text{(S1)}$$

$$Y = \frac{\int_{380\,nm}^{825\,nm} T(\lambda)P(\lambda)\,y(\lambda)\,d\lambda}{\int_{380\,nm}^{825\,nm} P(\lambda)\,y(\lambda)\,d\lambda} \quad \text{(S2)}$$

$$Z = \frac{\int_{380\,nm}^{825\,nm} T(\lambda)P(\lambda)\,z(\lambda)\,d\lambda}{\int_{380\,nm}^{825\,nm} P(\lambda)\,y(\lambda)\,d\lambda} \quad \text{(S3)}$$

where

$T(\lambda)$ is the transmittances of the device,

$P(\lambda)$ is the spectral irradiance of AM1.5G (100 mW cm^{-2}).

$x(\lambda)$, $y(\lambda)$, and $z(\lambda)$ are the color-matching functions that represent the red, green, and blue color sensations, respectively, of the CIE standard observer (Figure S20).

Further, XYZ tri-stimulus values were used to obtain the chromaticity coordinates of CIE 1931, as given below:[14]

$$x = \frac{X}{X+Y+Z} \quad \text{(S4)}$$

$$y = \frac{X}{X+Y+Z} \quad \text{(S5)}$$

According to the standard CIE 1976 (also called CIELUV), color coordinates (u,’ v’) can be obtained from XYZ tri-stimulus values as given below:[S14]

$$u' = \frac{4X}{X+15Y+3Z} = \frac{4x}{-2x+12y+3} \quad \text{(S6)}$$

$$v' = \frac{9X}{X+15Y+3Z} = \frac{9x}{-2x+12y+3} \quad \text{(S7)}$$

The color temperature has been used as a metric to characterize broadband light sources, measured in K. Regarding objects that do not produce light from a heated element, their color temperature can be characterized by the correlated color temperature (CCT), which is also measured in K.

McCamy’s formula was used to determine the CCT from the chromaticity coordinates, as given below:[S15]

$$CCT(K) = 449n^3 + 3525n^2 + 6823.3n + 5520.33 \quad \text{(S8)}$$

where $n = \frac{(x-0.3320)}{(0.1858-y)}$

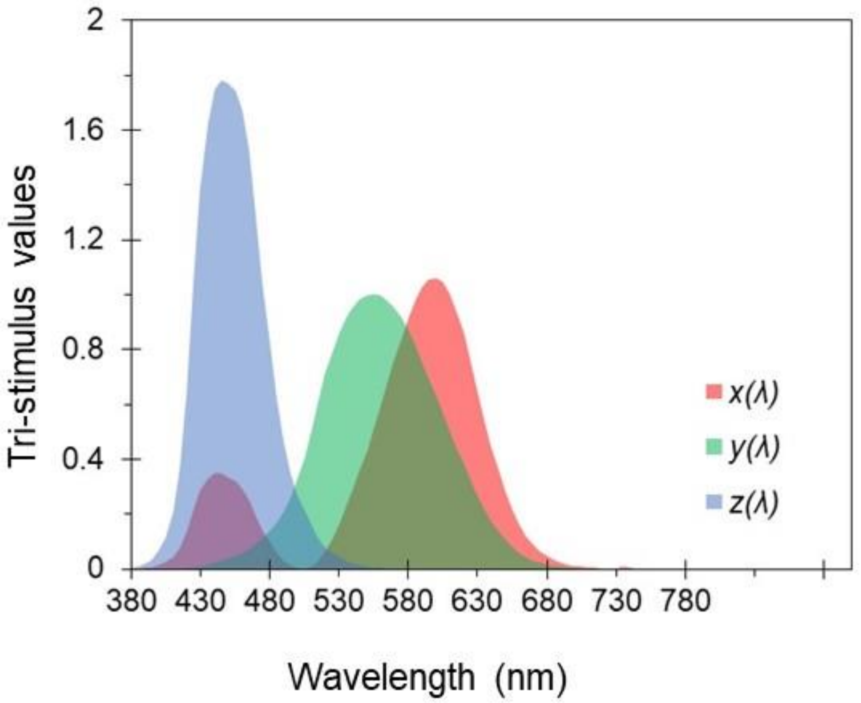

Figure S20. $x(\lambda)$, $y(\lambda)$, and $z(\lambda)$ are the color-matching functions representing the red, green, and blue color sensations, respectively, of the standard observer CIE.

Table S3. Summary of color analysis of the device with various angular lights. Here, AVT is the average visible transmittance, (X,Y,Z) represents the tri-stimulus values, (x, y) represents the chromaticity coordinates of CIE 1931, (u', v') represents the chromaticity coordinates of CIE 1931, n is the exponent in McCamy's formula, and CCT is the correlated color temperature.

Angle (°)	AVT (%)	*X*	*Y*	*Z*	Color coordinates					CCT (K)
					x	*y*	*u'*	*v'*	*n*	
90	31.36	33.891	31.489	6.898	0.469	0.436	0.257	0.538	-0.548	2766.3
85	39.13	42.149	39.291	9.339	0.464	0.433	0.256	0.536	-0.536	2808.0
80	39.40	42.490	39.567	9.364	0.465	0.433	0.256	0.536	-0.538	2801.3
70	39.37	42.537	39.536	9.266	0.466	0.433	0.256	0.536	-0.541	2788.8
60	39.09	42.344	39.254	9.066	0.467	0.433	0.257	0.537	-0.546	2771.2
45	38.24	41.608	38.399	8.631	0.469	0.433	0.259	0.537	-0.555	2741.0
40	37.12	40.573	37.279	8.142	0.472	0.434	0.260	0.538	-0.564	2711.4
35	36.57	40.030	36.726	7.943	0.473	0.434	0.260	0.538	-0.567	2701.6
25	35.25	38.728	35.400	7.462	0.475	0.434	0.262	0.538	-0.575	2676.7

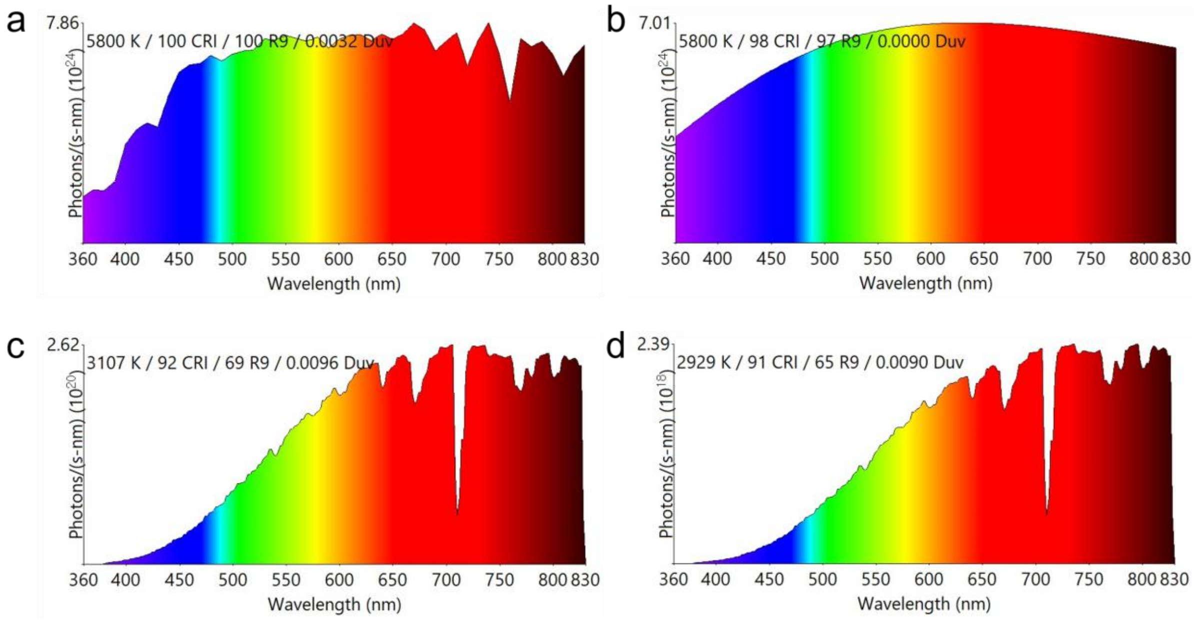

Figure S21. Photon flux as a function of photon wavelength to calculate color temperature (CCT), color rendering index (CRI), and CIELab parameters.

Table S4. Color analysis of daylight, blackbody spectrum, and AEW (at an incident angle of light).

Object details	CRI
Daylight(5800K)	100
Blackbody (5800K)	98
AEW-90°	92
AEW-85°	92
AEW-80°	92
AEW-70°	92
AEW-60°	92
AEW-45°	92
AEW-40°	91
AEW-35°	91
AEW-25°	91

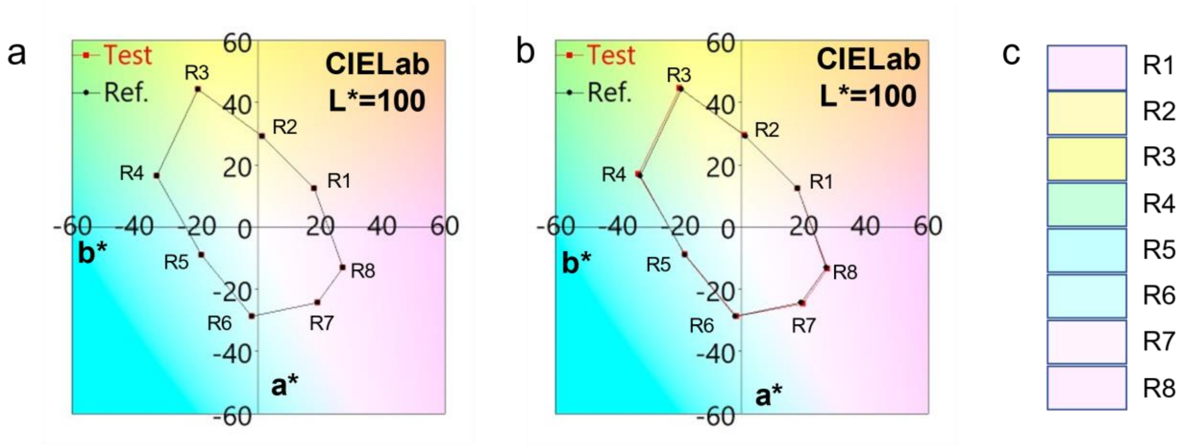

Figure S22. Color analysis. CIELab plot of (a) reconstituted daylight of 5800 K, (b) blackbody irradiance of 5800 K for L*=100. (c) Test color samples of R1-R8.

Table S5. CIELab parameters of active energy window, blackbody radiation of 5800 K, and reconstituted daylight of 5800 K. Here, L is lightness, a* is the degree of red/green, b* is the degree of blue/green, h* is hue angle in degrees, C* chroma, ΔL* is the shift in L* from the reference to the test, Δa* is the shift in a* from the reference to the test, Δb* is the shift in b* from the reference to the test, and ΔE* the total color difference. The estimation standard is referred to by the following. Beyond the perception (ΔE* <1), excellent color configuration (1< ΔE* <3, good configuration (3< ΔE* <6), and bad quality (ΔE* >10). The significance of ΔE* values is obtained from the ColorCalculator v7.77.

Colors	L* (Test)	a* (Test)	b* (Test)	h* (Test)	C* (Test)	L* (Ref)	a* (Ref)	b* (Ref)	h* (Ref)	C* (Ref)	ΔL*	Δa*	Δb*	ΔE*
**Active energy window**														
R1	63.5	19.0	15.4	39.0	24.4	63.6	19.5	15.7	38.9	25.0	-0.1	-0.5	-0.4	0.6
R2	62.0	5.6	27.4	78.4	27.9	61.9	5.4	29.8	79.7	30.3	0.0	0.2	-2.4	2.4
R3	62.3	-13.1	37.8	109.2	40.0	62.1	-14.0	41.8	108.6	44.1	0.2	0.9	-4.0	4.1
R4	59.4	-26.5	7.5	164.1	27.6	59.3	-28.3	10.8	159.1	30.3	0.1	1.8	-3.3	3.7
R5	60.4	-19.3	-13.1	214.1	23.3	60.4	-20.2	-12.6	212.0	23.9	0.1	0.9	-0.4	1.0
R6	59.4	-8.2	-30.1	254.8	31.2	59.4	-7.9	-31.2	255.9	32.2	-0.1	-0.3	1.1	1.1
R7	61.2	13.0	-20.2	302.9	24.0	61.4	14.5	-22.3	303.0	26.6	-0.2	-1.4	2.2	2.6
R8	64.2	24.9	-7.8	342.6	26.1	64.5	26.7	-9.1	341.1	28.2	-0.3	-1.8	1.3	2.3
**Blackbody radiation of 5800 K**														
Colors	L* (Test)	a* (Test)	b* (Test)	h* (Test)	C* (Test)	L* (Ref)	a* (Ref)	b* (Ref)	h* (Ref)	C* (Ref)	ΔL*	Δa*	Δb*	ΔE*
R1	61.8	18.1	12.3	34.3	21.9	61.7	18.1	12.3	34.2	21.9	0.1	-0.1	0.0	0.1
R2	60.9	0.9	29.6	88.2	29.6	60.8	1.3	29.2	87.5	29.2	0.1	-0.3	0.4	0.5
R3	62.1	-19.9	44.7	114.0	48.9	62.1	-19.4	44.3	113.6	48.4	0.0	-0.5	0.4	0.6
R4	61.0	-33.2	17.0	152.8	37.3	61.0	-32.5	16.4	153.3	36.4	-0.1	-0.6	0.7	0.9
R5	62.1	-18.3	-8.8	205.7	20.3	62.2	-18.2	-9.0	206.3	20.3	-0.1	-0.1	0.2	0.2
R6	61.2	-1.6	-28.8	266.8	28.8	61.2	-2.0	-28.7	266.1	28.8	-0.1	0.3	-0.1	0.4
R7	61.1	19.7	-24.8	308.5	31.6	61.1	19.1	-24.3	308.2	30.9	0.0	0.6	-0.5	0.8
R8	63.0	27.7	-13.4	334.2	30.8	63.0	27.4	-13.0	334.5	30.3	0.0	0.4	-0.4	0.5
**Reconstituted daylight of 5800 K**														
Colors	L* (Test)	a* (Test)	b* (Test)	h* (Test)	C* (Test)	L* (Ref)	a* (Ref)	b* (Ref)	h* (Ref)	C* (Ref)	ΔL*	Δa*	Δb*	ΔE*
R1	61.7	18.1	12.3	34.2	21.9	61.7	18.1	12.3	34.2	21.9	0.0	0.0	0.0	0.0
R2	60.8	1.3	29.2	87.5	29.2	60.8	1.3	29.2	87.5	29.2	0.0	0.0	0.0	0.0
R3	62.1	-19.4	44.3	113.6	48.4	62.1	-19.4	44.3	113.6	48.4	0.0	0.0	0.0	0.0
R4	61.0	-32.5	16.4	153.3	36.4	61.0	-32.5	16.4	153.3	36.4	0.0	0.0	0.0	0.0
R5	62.2	-18.2	-9.0	206.3	20.3	62.2	-18.2	-9.0	206.3	20.3	0.0	0.0	0.0	0.0
R6	61.2	-2.0	-28.7	266.1	28.8	61.2	-2.0	-28.7	266.1	28.8	0.0	0.0	0.0	0.0
R7	61.1	19.1	-24.3	308.2	30.9	61.1	19.1	-24.3	308.2	30.9	0.0	0.0	0.0	0.0
R8	63.0	27.4	-13.0	334.5	30.3	63.0	27.4	-13.0	334.5	30.3	0.0	0.0	0.0	0.0

Supporting References:

[S1] D. Ban, M. Patel, T. T. Nguyen, J. Kim, *Adv. Electron. Mater.* **2019**, *5*, 1900348.

[S2] T. T. Nguyen, M. Patel, J.-W. Kim, W. Lee, J. Kim, *J. Alloys Compd.* **2020**, *816*, 152602.

[S3] S. Abbas, M. Kumar, D. W. Kim, J. Kim, *Small* **2019**, *15*, 1804346.

[S4] P. Heinstein, C. Ballif, L. E. Perret-Aebi, *Green* **2013**, *3*, 125.

[S5] E. Andenæs, B. P. Jelle, K. Ramlo, T. Kolås, J. Selj, S. E. Foss, *Sol. Energy* **2018**, *159*, 318.

[S6] B. Marion, R. Schaefer, H. Caine, G. Sanchez, *Sol. Energy* **2013**, *97*, 112.

[S7] P.-O. A. Borrebæk, B. P. Jelle, Z. Zhang, *Sol. Energy Mater. Sol. Cells* **2020**, *206*, 110306.

[S8] M. van Noord, T. Landelius, S. Andersson, *Energies* **2021**, *14*, 1574.

[S9] N. Heidari, J. Gwamuri, T. Townsend, J. M. Pearce, *IEEE J. Photovoltaics* **2015**, *5*, 1680.

[S10] L. Burnham, D. Riley, B. Walker, J. M. Pearce, in *2019 IEEE 46th Photovolt. Spec. Conf.*, IEEE, **2019**, pp. 1320–1327.

[S11] M. Köntges, S. Kurtz, C. E. Packard, U. Jahn, K. Berger, K. Kato, T. Friesen, H. Liu, M. Van Iseghem, *Review of Failures of Photovoltaic Modules - IEA-PVPS T13-01:2014 : Https://Iea-Pvps.Org/Wp-Content/Uploads/2020/01/IEA-PVPS_T13-01_2014_Review_of_Failures_of_Photovoltaic_Modules_Final.Pdf*, **2014**.

[S12] S. Kurtz, *Photovoltaic Module Reliability Workshop 2011: Https://Www.Nrel.Gov/Docs/Fy11osti/51121.Pdf*, **2013**.

[S13] C. J. Traverse, R. Pandey, M. C. Barr, R. R. Lunt, *Nat. Energy* **2017**, *2*, 849.

[S14] D. Han, "Understanding CIE1931 and CIE 1976," **2019**.

[S15] T. A. O. SOLUTIONS, *Intelligent Opto Sensor Designer's Notebook*, **2009**.
